# Lorentzian length spaces

**DOI:** 10.1007/s10455-018-9633-1

**Published:** 2018-10-05

**Authors:** Michael Kunzinger, Clemens Sämann

**Affiliations:** Faculty of Mathematics, University of Vienna, Vienna, Austria

**Keywords:** Length spaces, Lorentzian length spaces, Causality theory, Synthetic curvature bounds, Triangle comparison, Metric geometry, 53C23, 53C50, 53B30, 53C80

## Abstract

We introduce an analogue of the theory of length spaces into the setting of Lorentzian geometry and causality theory. The rôle of the metric is taken over by the time separation function, in terms of which all basic notions are formulated. In this way, we recover many fundamental results in greater generality, while at the same time clarifying the minimal requirements for and the interdependence of the basic building blocks of the theory. A main focus of this work is the introduction of synthetic curvature bounds, akin to the theory of Alexandrov and CAT(*k*)-spaces, based on triangle comparison. Applications include Lorentzian manifolds with metrics of low regularity, closed cone structures, and certain approaches to quantum gravity.

## Introduction

Metric geometry, and in particular the theory of length spaces, is a vast and very active field of research that has found applications in diverse mathematical disciplines, such as differential geometry, group theory, dynamical systems and partial differential equations. It has led to identifying the “metric core” of many results in differential geometry, to clarifying the interdependence of various concepts, and to generalizations of central notions in the field to low regularity situations. In particular, the synthetic approach to curvature bounds in the theory of Alexandrov spaces and CAT(*k*)-spaces has turned out to be of fundamental importance (cf., e.g., [3, 5, 42]).

The purpose of this work is to lay the foundations for a synthetic approach to Lorentzian geometry that is similar in spirit to the theory of length spaces and that, in particular, allows one to introduce curvature bounds in this general setting. The motivation for our approach is very similar to that of metric geometry: ideally, it should identify the minimal requirements for and the logical dependence among fundamental results of Lorentzian geometry and causality theory, and in this way separate the essential concepts from various derived notions. Based on this, one may extend the validity of these results to their natural maximal range, in particular to settings where the Lorentzian metric is no longer differentiable, or even to situations where there is only a causal structure not necessarily induced by a metric. Again in parallel to the case of metric geometry, appropriate notions of synthetic (timelike or causal) curvature bounds based on triangle comparison occupy a central place in this development, leading to a minimal framework for Lorentzian comparison geometry. In the smooth case, triangle comparison methods were pioneered by Harris [24] for the case of timelike triangles in Lorentzian manifolds and by Alexander and Bishop [1] for the general semi-Riemannian case and triangles of arbitrary causal character. The notions introduced in this paper are designed to be compatible with these works, while at the same time introducing curvature bounds even to settings where there is no curvature tensor available (due to low differentiability of the metric or even the absence thereof). On the one hand, this makes it possible to establish well-known results from metric geometry also in the Lorentzian setting (e.g., non-branching of maximal curves in spaces with timelike curvature bounded below, cf. Theorem 4.12). On the other hand, it provides a new perspective on genuinely Lorentzian phenomena, like the push-up principle for causal curves, which in the present context appears as a consequence of upper causal curvature bounds (see Sect. 4.5).

The rôle of the metric of a length space in the current framework will be played by the time separation function $\tau$, which will therefore be our main object of study. It is closely linked to the causal structure of Lorentzian manifolds, and in fact for strongly causal spacetimes it completely determines the metric by a classical result of Hawking, cf. the beginning of Sect. 2.3. We will express all fundamental notions of Lorentzian (pre-)length spaces (such as length and maximality of curves, curvature bounds, etc.) in terms of $\tau$. It turns out that this provides a satisfactory description of much of standard causality theory and recovers many fundamental results in greater generality.

Apart from the intrinsic interest in a Lorentzian analogue of metric geometry, a main motivation for this work is the necessity to consider Lorentzian metrics of low regularity. This need is apparent both from the PDE point of view on general relativity, i.e., the initial value problem for the Einstein equations, and from physically relevant models. In fact, the standard local existence result for the vacuum Einstein equations [47] assumes the metric to be of Sobolev regularity $H^s_\text {loc}$ (with $s>\frac{5}{2}$). Recently, the regularity of the metric has been lowered even further (e.g., [28]). In many cases, spacetimes describing physically relevant situations require certain restrictions on the regularity of the metric. In particular, modeling different types of matter in a spacetime may lead to a discontinuous energy-momentum tensor, and thereby via the Einstein equations to metrics of regularity below ${\mathcal {C}}^2$ [34, 39]. Prominent examples are spacetimes that model the inside and outside of a star or shock waves. Physically relevant models of even lower regularity include spacetimes with conical singularities and cosmic strings (e.g., [55, 56]), (impulsive) gravitational waves (see, e.g., [43, 23, Ch. 20] and [45, 46, 52] for more recent works), and ultrarelativistic black holes (e.g., [2]).

There has in fact recently been a surge in activity in the field of low regularity Lorentzian geometry and mathematical relativity. Some main trends in this branch of research are the studies in geometry and causality theory for Lorentzian metrics of low regularity (see [12, 48] for results on continuous metrics and [29, 30, 36] for the ${\mathcal {C}}^{1,1}$-setting). Cone structures on differentiable manifolds are another natural generalization of smooth Lorentzian geometry, and several recent fundamental works have led to far-reaching extensions of causality theory, see [10, 18, 37]. Another line of research concerns the extension of the classical singularity theorems of Hawking and Penrose to minimal regularity [19, 31, 32]. In fact, both spacetimes with metrics of low regularity and closed cone structures of certain types provide large natural classes of examples within the theory of Lorentzian length spaces (cf. Sect. 5). Finally, we mention the recent breakthroughs concerning the ${\mathcal {C}}^0$-inextendibility of spacetimes, pioneered by Sbierski [49] in the case of the Schwarzschild solution to the Einstein equations, followed by further investigations by various authors, cf. [14, 21, 22]. As we shall point out repeatedly throughout this work, there are close ties between these works and the theory of Lorentzian length spaces. More specifically, the follow-up work [20]

- introduces a notion of extendibility for Lorentzian (pre-)length spaces that reduces to isometric embeddability in the particular case of spacetimes,
- gives a characterization of timelike completeness in terms of the time separation function,
- shows that timelike completeness in this sense implies inextendibility even in this general setting, and
- for the first time, relates inextendibility to the occurrence of (synthetic) curvature singularities.

This complements and extends the works cited above in several directions. In particular, both the original spacetime and the extension are now allowed to be of low regularity and indeed also non-manifold extensions can be considered.

There have been several approaches to give a synthetic or axiomatic description of (parts of) Lorentzian geometry and causality. However, except the work of Busemann [11] they were not in the spirit of metric geometry and length spaces. Moreover, triangle comparison was never used in these settings. In particular, Busemann—a pioneer of length spaces—introduced so-called *timelike spaces* in [11], but his approach was too restrictive to even capture all smooth (globally hyperbolic) Lorentzian manifolds. Another closely related work is due to Kronheimer and Penrose [27], who studied the properties and interdependences of the causal relations in complete generality. Similar in spirit, Sorkin and Woolgar [54] established that using order-theoretic and topological methods one can extend specific results from causality theory to spacetimes with continuous metrics. On the other hand, Martin and Panangaden showed in [38] how one recovers a spacetime from just the causality relations in an order-theoretic manner, thereby indicating applications to quantum gravity. For this reason, Lorentzian (pre-)length spaces might provide a fundamental framework to approaches to quantum gravity, as outlined in Sect. 5.3. In a series of works, Borchers and Sen [7–9] describe an approach to causality via an axiomatic notion of *light rays* corresponding to null geodesics. Finally, and very relevant to the goals of this paper, Sormani and Vega [53] have recently introduced a metric structure on (smooth) spacetimes via what they call a null distance function. It is defined as the infimum over *null lengths*, which in turn are total variations of a time function along concatenations of (future or past-directed) causal curves. The null distance provides a conformally invariant pseudo-metric, and under some natural assumptions on the spacetime even a definite distance function inducing the manifold topology. This leads to an alternative starting point for studying metric geometry in the Lorentzian setting.

The plan of the paper is as follows: In the remainder of this section, we fix some basic notions used throughout this work. Section 2 introduces the fundamental causal and chronological relations, the definition of Lorentzian pre-length spaces in terms of a lower semicontinuous time separation function, and the associated topological notions. It also includes the fundamentals of causal curves. To obtain a satisfying causality theory close to that of smooth spacetimes, more structure is required. This leads, in Sect. 3, to the notion of Lorentzian length space. We show that in this setting, most of standard causality theory remains intact, including limit curve theorems, push-up principles for causal curves, (a significant part of) the causal ladder and the Avez–Seifert theorem for globally hyperbolic spaces. We also introduce a synthetic notion of regularity for Lorentzian pre-length spaces. Section 4 is devoted to synthetic curvature bounds in terms of triangle comparison. Time separation in timelike and causal geodesic triangles is compared with corresponding quantities in Lorentzian model spaces of constant curvature. In the smooth case, this is compatible with the Toponogov-type results of Alexander and Bishop [1]. Applications include a non-branching theorem for timelike curves in spaces with timelike curvature bounded below (Theorem 4.12) and a new interpretation of length-increasing push-up in terms of upper bounds on causal curvature (Theorem 4.18). We then go on to defining synthetic curvature singularities and show that the central singularity of the interior Schwarzschild solution can be detected via timelike triangle comparison. The final Sect. 5 provides an in-depth study of probably the most important class of examples, namely continuous Lorentzian metrics. Apart from identifying Lorentzian length spaces among continuous spacetimes, we also derive several new results on continuous causality theory in this section. In addition, we consider closed cone structures following the recent fundamental work of Minguzzi [37] and give a brief outlook on potential applications in certain approaches to quantum gravity, namely causal Fermion systems and the theory of causal sets.

To conclude this introduction, we introduce some basic notions and fix notations. For terminology from or motivated by causality theory, we will follow the standard texts [4, 13, 40, 41], see also [12] for the case of continuous Lorentzian metrics. We will usually only formulate the future-directed case of our results, with the understanding that there always is an analogous past-directed statement.

Among the main applications of the theory developed here will be spacetimes (*M*, *g*), where *M* is a differentiable manifold and *g* is a continuous or smooth Lorentzian metric. We always assume that (*M*, *g*) is time-oriented (i.e., there is a continuous timelike vector field on *M*) and we fix a smooth (without loss of generality) complete Riemannian metric *h* on *M*, denoting the induced metric by $d^h$.

Recall that for (continuous) Lorentzian metrics $g,\ {\hat{g}}$ on *M*, $g\prec {\hat{g}}$ is defined as the property that the light cones of ${\hat{g}}$ are strictly wider than those of *g*, i.e., if a nonzero vector is causal for *g* then it is timelike for ${\hat{g}}$. Additionally, we also use the non-strict version, i.e., we mean by $g\preceq {\hat{g}}$ that every *g*-causal vector is causal for ${\hat{g}}$.

Causal curves in spacetimes are locally Lipschitz continuous curves $\gamma :I\rightarrow M$ with $g({{\dot{\gamma }}},{{\dot{\gamma }}})\le 0$ almost everywhere. Analogously for timelike and null curves and their time orientation.

## Basics

### Causal relations

We start our analysis by introducing a slightly more general notion of causal spaces, as compared to [27].

#### Definition 2.1

Let *X* be a set with a reflexive and transitive relation $\le$ (a *pre-order*) and $\ll$ a transitive relation contained in $\le$ (i.e., $\ll \,\subseteq \,\le$, or more explicitly: If $x\ll y$ then $x\le y$). Then, $(X,\ll ,\le )$ is called a *causal space*. We write $x<y$ if $x\le y$ and $x\ne y$.

#### Example 2.2

Any spacetime with a continuous metric and the usual causal relations (e.g., $x\ll y$ if there is a future-directed timelike curve from *x* to *y*) is a causal space. Contrary to [27], we do not require any causality condition to hold. Thus, also the Lorentz cylinder $M=S^1_1\times \mathbb {R}$ [41, Example 5.35] is an example, and in this case $\ll \ =\ \le \ = M\times M$. We will study spacetimes with continuous metrics in greater detail in Sect. 5.1.

#### Definition 2.3

(*Futures and pasts*) For $x\in X$ define

(i) $$I^+(x):=\{y\in X: x\ll y\}$$ and $$I^-(x):=\{y\in X: y\ll x\}$$,
(ii) $$J^+(x):=\{y\in X: x\le y\}$$ and $$J^-(x):=\{y\in X: y\le x\}$$.

### Topologies on causal spaces

On a causal space, one can define two natural topologies as follows. Let $(X,\ll ,\le )$ be a causal space. For $x,y\in X$ define $I(x,y):=I^+(x)\cap I^-(y)\subseteq X$.

#### Definition 2.4

(*Topologies on* $(X,\ll ,\le )$) Let $(X,\ll ,\le )$ be a causal space.

(i) Define a topology ${\mathcal {A}}$ on *X* by using $S:= \{I(x,y): x,y\in X\}$ as a subbase. We call this topology the *Alexandrov topology* on *X* with respect to $\ll$.
(ii) Define a topology ${\mathcal {I}}$ on *X* by using $P:=\{I^\pm (x):x\in X\}$ as a subbase. We call this topology the *chronological topology* on *X*.

#### Example 2.5

**Fig. 1:**
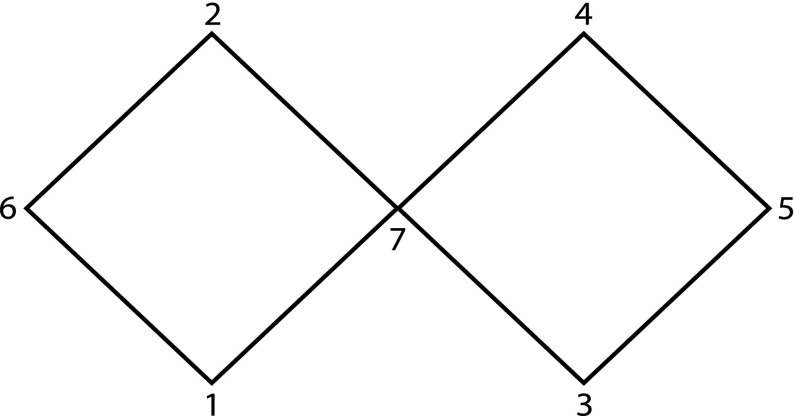
Graph for Example 2.5

(*S* *and* *P* *are not bases for topologies*) In general, the sets *S* and *P* do not form bases for topologies as this simple example shows. Let $X=\{1,\ldots ,7\}$ and let $\ll$ be given via the graph (Fig. 1) (e.g., $1\ll 7$ etc.). This is a transitive and irreflexive relation.

Then:

(i) The point 1 is not covered by any *I*(*x*, *y*) for $x,y\in X$, thus *S* does not cover *X*.
(ii) Although *P* does cover *X*, it does not have the second property required for a basis: $I^+(1)\cap I^-(2) = \{6,7\}$, but there is no $x\in X$ such that $7\in I^\pm (x)\subseteq \{6,7\}$.

#### Remark 2.6

Note that in general the future and past $I^\pm (x)$ is not open with respect to ${\mathcal {A}}$. As an example, take $X=\{1,2\}$ with $\ll :=\{(1,2)\}$, then $S=\{\emptyset \}$ hence ${\mathcal {A}}=\{\emptyset , X\}$ is the trivial topology and $I^+(1)=\{2\}$.

#### Proposition 2.7

The chronological topology ${\mathcal {I}}$ is finer than the Alexandrov topology ${\mathcal {A}}$. Consequently, a map from *X* into a topological space *Y* that is ${\mathcal {A}}$-continuous is also ${\mathcal {I}}$-continuous. In particular, for $Y=[0,\infty ]$ the same holds for semicontinuous maps.

#### Proof

The Alexandrov topology ${\mathcal {A}}$ is the coarsest topology containing *S* and thus since ${\mathcal {I}}$ contains *S* we have ${\mathcal {A}}\subseteq {\mathcal {I}}$. $\square$

The example in 2.6 shows that in general ${\mathcal {I}}$ is strictly finer than ${\mathcal {A}}$. In that case ${\mathcal {A}}=\{\emptyset , X\}$ and ${\mathcal {I}}$ is the discrete topology.

In what follows we will not work directly with one of these topologies, but mainly use them for comparison. Instead, we will assume that the causal space *X* also comes with the structure of a metric space.

### Lorentzian pre-length spaces

We now introduce the central object of our study, namely the time separation function $\tau$, in terms of which all subsequent concepts will be formulated. While it is evident that in the smooth setting $\tau$ is closely linked to the causal structure, it is worth noting that by a classical result due to Hawking et al. [25] for strongly causal spacetimes it in fact completely determines the metric, (cf. [40, Prop. 3.34] or [4, Thm. 4.17]).

#### Definition 2.8

Let $(X,\ll ,\le )$ be a causal space and *d* a metric on *X*. Let $\tau :X\times X\rightarrow [0,\infty ]$ be a lower semicontinuous map (with respect to the metric topology induced by *d*) that satisfies

1 $$\begin{aligned} \tau (x,z)\ge \tau (x,y) + \tau (y,z), \end{aligned}$$

for all $x,y,z\in X$ with $x\le y\le z$. Moreover, suppose that $\tau (x,y)=0$ if $x\nleq y$ and $\tau (x,y)>0 \Leftrightarrow x\ll y$. Then, $(X,d,\ll ,\le ,\tau )$ is called a *Lorentzian pre-length space*, and $\tau$ is called the *time separation function*.

Since we now use the metric *d* on *X*, all topological notions refer to the metric topology ${\mathcal {D}}$ induced by *d*, unless specified otherwise.

#### Remark 2.9

It would be logically possible to introduce Lorentzian pre-length spaces based only on a set endowed with a pre-order $\le$ and then *define* the timelike relation $\ll$ via $x\ll y :\Leftrightarrow \tau (x,y)>0$. However, we prefer to view this condition as a form of compatibility between the time separation function and the causal space. For an example where this compatibility is violated see Example 5.2.

#### Lemma 2.10

(Push-up) Let $(X,d,\ll ,\le ,\tau )$ be a Lorentzian pre-length space and let $x,y,z\in X$ with $x\le y\ll z$ or $x\ll y\le z$. Then, $x\ll y$.

#### Proof

Let $x\le y\ll z$ or $x\ll y\le z$. Then, $\tau (x,z)\ge \tau (x,y)+\tau (y,z)>0$, which implies $x\ll z$. $\square$

#### Example 2.11

Let (*M*, *g*) be a smooth spacetime with its canonical causal relations $\ll$ and $\le$. Then, by Example 2.2$(M,\ll ,\le )$ is a causal space. The (classical) time separation function $\tau$ is lower semicontinuous with respect to the manifold topology [41, Lemma 14.17], which is induced by any Riemannian metric *h* on *M* and its associated metric $d^h$. Moreover, $\tau$ satisfies the reverse triangle inequality (1) and $\tau (p,q)>0 \Leftrightarrow p\ll q$, thus $(M,d^h, \ll ,\le ,\tau )$ is a Lorentzian pre-length space. Note that, in general, for a spacetime with a continuous metric the time separation function $\tau$ need not be lower semicontinuous, cf. Example 5.2.

We will establish some basic facts about Lorentzian pre-length spaces below.

#### Lemma 2.12

Let $(X,d,\ll ,\le ,\tau )$ be a Lorentzian pre-length space and $x\in X$. Then, $I^\pm (x)$ is open.

#### Proof

We establish this fact only for $I^+(x)$, the case for $I^-(x)$ works in complete analogy. Let $y\in I^+(x)$, so $x\ll y$ and thus $\tau (x,y)>0$. By the lower semicontinuity of $\tau (x,.)$, there is a neighborhood *U* of *y* such that $\tau (x,z)>\frac{\tau (x,y)}{2}>0$ for all $z\in U$. Consequently, $x\ll z$ and thus $y\in U\subseteq I^+(x)$. $\square$

#### Proposition 2.13

Let $(X,d,\ll ,\le ,\tau )$ be a Lorentzian pre-length space. Then, the relation $\ll$ is an open subset of $X\times X$.

#### Proof

Clearly, $\ll = \{(x,y)\in X\times X: \tau (x,y)>0\}$, which is open by the lower semicontinuity of $\tau$. $\square$

#### Proposition 2.14

Let $(X,d,\ll ,\le ,\tau )$ be a Lorentzian pre-length space. Then, for every $x\in X$ either $\tau (x,x)=0$ or $\tau (x,x)=\infty$. Moreover, if $\tau (x,y)\in (0,\infty )$, then $\tau (y,x)=0$.

#### Proof

Let $x\in X$ with $\tau (x,x)<\infty$. By the reflexivity of $\le$, we have $x\le x \le x$ and thus by the reverse triangle inequality we have $\tau (x,x) + \tau (x,x) \le \tau (x,x)$, which implies that $\tau (x,x)\le 0$ and so $\tau (x,x)=0$. Finally, let $0<\tau (x,y)<\infty$ and suppose that $\tau (y,x)>0$. Then, $x\ll y\ll x$, which implies $x\ll x$ and by the above $\tau (x,x)=\infty$. But then $\tau (x,x)+\tau (x,y) \le \tau (x,y)<\infty$ gives a contradiction. $\square$

#### Remark 2.15

The above proposition shows that a set *X* with a transitive relation $\ll$ that is not irreflexive (i.e., $x\ll x$ for some *x*) cannot have a finite valued time separation function with respect to $\ll$ (and any transitive and reflexive relation $\le$ on *X* containing $\ll$). In fact, $x\ll x$ implies $\tau (x,x)>0$, but by the above $\tau (x,x)\le 0$, if $\tau$ were finite valued.

#### Example 2.16

A finite directed graph (*V*, *E*) can be given the structure of a Lorentzian pre-length space. Here, *X* is any finite set, $V\subseteq X$ is the set of vertices, and $E\subseteq V\times V$ is the (directed) set of edges, i.e., $(x,y)\in E$ if and only if there is an edge from *x* to *y*. Now define $x\ll y$ if $(x,y)\in E$ and $x\le y$ if $x\ll y$ or $x=y$. This gives a causal space $(V,\ll ,\le )$, where $\ll$ is irreflexive if and only if (*V*, *E*) is a directed acyclic graph. Define $\tau (x,y):= \sup \{ |C|: C$ a directed subgraph from *x* to $y \}$, if such a subgraph exists and |*C*| denotes its (finite) cardinality; otherwise set $\tau (x,y):=0$. Since the only metrizable topology on a finite set is the discrete topology, we let *d* be the discrete metric on *V*. Thus, $\tau$ is continuous and it satisfies the reverse triangle inequality (1). This yields that $(V,d,\ll ,\le ,\tau )$ is a Lorentzian pre-length space.

Note that since the topology is discrete, the space is totally disconnected, and hence, there are no causal curves (cf. Definition 2.18). Causal curves will be essential in the development of the theory of Lorentzian length spaces, and it will be a requirement on such spaces that causally related points can be connected by a (non-constant) continuous causal curve (cf. Definition 3.1). This rules out finite and, in fact, countable Lorentzian length spaces, as the only metrizable topology in these cases is totally disconnected. Thus, the situation is the same as for length spaces—they are path connected and hence cannot be countable.

This example is closely related to the theory of *causal sets*, an approach to quantum gravity, see Sect. 5.3.

### Topologies on Lorentzian pre-length spaces

We want to relate the two natural topologies ${\mathcal {I}}$ and ${\mathcal {A}}$ to the given metric topology ${\mathcal {D}}$.

One can see from the proof of Lemma 2.12 that the only property used for the topology is that $\tau$ be semicontinuous with respect to it. Thus, if one has a function $\tau :X\times X\rightarrow [0,\infty ]$ that satisfies the properties of a time separation function (as in Definition 2.8) except that it is lower semicontinuous with respect to ${\mathcal {A}}$ (and not necessarily with respect to *d*), then $I^\pm (x)$ is ${\mathcal {A}}$-open for every $x\in X$. Consequently, ${\mathcal {A}}$ is finer than ${\mathcal {I}}$ and so by Proposition 2.7 we have ${\mathcal {I}}={\mathcal {A}}$. On the other hand, the metric topology ${\mathcal {D}}$ is finer than both these topologies since by the extension of Lemma 2.12 mentioned above $I^\pm (x)$ and $I^+(x)\cap I^-(y)$ are ${\mathcal {D}}$-open for every $x,y\in X$. This yields that

(i) in an “${\mathcal {A}}$-Lorentzian pre-length space” (i.e., $\tau$ lower semicontinuous with respect to ${\mathcal {A}}$) we have ${\mathcal {A}}={\mathcal {I}}$ and thus
(ii) every “${\mathcal {A}}$-Lorentzian pre-length space” is also an “${\mathcal {I}}$-Lorentzian pre-length space”, which is also a Lorentzian pre-length space (since if $\tau$ is lower semicontinuous with respect to ${\mathcal {I}}$, then it is lower semicontinuous with respect to ${\mathcal {D}}$).

Summing up, by using the additional freedom via the metric *d* we (possibly) enlarge the number of Lorentzian pre-length spaces, as can be seen from the example below.

#### Example 2.17

In light of Remark 2.6 and the above, we know that there cannot be a time separation function $\tau$ on $(X=\{1,2\},\ll =\{(1,2)\},\le =X\times X)$ that is lower semicontinuous with respect to ${\mathcal {A}}$. This can be also seen directly from the fact that a prospective time separation function $\tau$ would need to satisfy $\tau (1,1)=\tau (2,2) = \tau (2,1)=0$ and $\tau (1,2)>0$. However, the only ${\mathcal {A}}$-open sets in $X\times X$ are $\emptyset$ and $X\times X$ and thus $\{(x,y)\in X\times X: \tau (x,y)>0\} = \{(1,2)\}$ is not ${\mathcal {A}}$-open (which would be required if $\tau$ were ${\mathcal {A}}$-lower semicontinuous).

### Causal curves

At this point, we introduce *timelike*, *causal* and *null curves*, which will be defined via the corresponding relations. One has to note that even in the case of a smooth spacetime the class of timelike or causal curves obtained in this way differs in general from the class of timelike or causal curves defined in the usual way (via the causal character of the tangent vector). If one assumes additionally that a smooth spacetime is strongly causal then the classes of causal curves are the same. On the other hand, the classes of timelike curves are still different in general. This will be discussed in more detail in Example 2.20. It will, however, not be an issue since we are mainly interested in the length of curves and strong causality ensures that the lengths are unchanged, see Proposition 2.32. A causal curve is non-constant by definition but it could be constant on some interval, contrary to causal curves in a spacetime. However, even such curves can be parametrized with respect to *d*-arclength, see [42, Prop. 1.2.2, Cor. 1.2.6].

#### Definition 2.18

Let $I\subseteq \mathbb {R}$ be an interval. A non-constant curve $\gamma :I\rightarrow X$ is called *future-directed causal (timelike)* if $\gamma$ is locally Lipschitz continuous (with respect to *d*) and for all $t_1,t_2\in I$, $t_1<t_2$ we have that $\gamma (t_1)\le \gamma (t_2)$ ($\gamma (t_1)\ll \gamma (t_2)$). Furthermore, a future-directed causal curve is called *null* if no two points on the curve are related with respect to $\ll$. Analogous notions are introduced for *past-directed* curves.

Note that if *d* is the discrete metric on *X*, then there are no causal curves, since in this case (*X*, *d*) is totally disconnected and any continuous curve is constant.

#### Lemma 2.19

Let $\gamma :[a,b]\rightarrow X$ be a causal curve, then $\gamma$ is Lipschitz continuous and *d*-rectifiable.

#### Proof

Since the domain of $\gamma$ is compact, local Lipschitz continuity implies Lipschitz continuity, which in turn implies *d*-rectifiability. $\square$

Let us now investigate the relationship of the different notions of causal and timelike curves for the case of continuous or smooth spacetimes.

#### Example 2.20

Let (*M*, *g*) be the Lorentz cylinder (cf. Example 2.2), then since $\ll \ =\ \le \ = M\times M$, every non-constant, locally Lipschitz continuous curve is timelike and causal in the sense of Definition 2.18. Consequently, there are no null curves (again in the sense of Definition 2.18). Thus, this class of timelike and causal curves is larger than the usual class of timelike and causal curves. We will clarify the precise relationship in Lemma 2.21.

In the following result (and thereafter), when comparing the notions of causal curves in the present setting with the standard definition in spacetimes, it will always be understood that parametrizations are chosen in which the respective curves are never locally constant (cf. [3, Ex. 2.5.3]).

#### Lemma 2.21

Let (*M*, *g*) be a spacetime with a continuous metric. For clarity, we call timelike and causal curves in the sense of Definition 2.18 *R-timelike* and *R-causal*, respectively.

(i) A causal/timelike curve is an R-causal/R-timelike curve.
(ii) If (*M*, *g*) is a smooth, strongly causal [41, Def. 14.11] spacetime, then the notions of causal, R-causal and continuous causal curves [57, p. 192f.] coincide.
(iii) If (*M*, *g*) is a smooth, strongly causal spacetime, then a locally Lipschitz continuous curve is an R-timelike curve if and only if it is a continuous timelike curve [57, p. 192f.]. However, in general the tangent vector of such a curve is only causal almost everywhere, as can be seen from Example 2.22.

#### Proof

Let $\gamma :I\rightarrow M$ be a locally Lipschitz continuous curve.

(i) Without loss of generality, let $\gamma$ be a future-directed causal/timelike curve. Let $t_1,t_2\in I$ with $t_1<t_2$, then $\gamma$ is a future-directed causal/timelike curve from $\gamma (t_1)$ to $\gamma (t_2)$; hence, $\gamma$ is R-causal/R-timelike.
(ii) Without loss of generality, let $\gamma$ be a future-directed R-causal curve and let $t_0\in I$. Let *U* be a convex neighborhood of $\gamma (t_0)$, and let $V\subseteq U$ be a neighborhood of $\gamma (t_0)$ such that every causal curve with endpoints in *V* is contained in *U*. Let $t_1,t_2\in I$ such that $\gamma (t_1),\gamma (t_2)\in V$; hence, $\gamma ([t_1,t_2])\subseteq U$ and consequently $\gamma (t_2)\in J^+(\gamma (t_1),U)$, where $J^+(\gamma (t_1), U)$ denotes the set of all points which can be reached from $\gamma (t_1)$ by a Lipschitz continuous future-directed causal curve contained in *U*. This is the same set as constructed via piecewise smooth causal curves (cf. [33, Prop. 2.3], [44, Fig. 44], and [13, Cor. 2.4.11] for the Lipschitz case). This establishes that $\gamma$ is a continuous causal curve. Moreover, by [41, Lemma 14.2(1)], $\overrightarrow{\gamma (t_1)\gamma (t_2)}\,=\,\mathrm {exp}_{\gamma (t_1)}^{-1}(\gamma (t_2))$ is causal. Consequently, at any $t_0$ where $\gamma$ is differentiable,
  $$\begin{aligned} {{\dot{\gamma }}}(t_0) = \lim _{h\rightarrow 0}\frac{1}{h} \overrightarrow{\gamma (t_0)\gamma (t_0+h)} \end{aligned}$$
  is causal, so $\gamma$ is a future-directed causal curve.
(iii) That any R-timelike $\gamma$ is a future-directed continuous timelike curve again follows as in point (i) by applying [13, Cor. 2.4.11]. The converse is clear.

$$\square $$

In fact, as we shall see in Proposition 5.9, the conclusion of Lemma 2.21 (ii) can be extended to continuous spacetimes.

#### Example 2.22

Denote by $\mathbb {R}^n_1$ the *n*-dimensional Minkowski spacetime and define $\gamma :\mathbb {R}\rightarrow \mathbb {R}^3_1$ by $\gamma (t)=(t,\cos (t),\sin (t))$ for $t\in \mathbb {R}$. Then, $\gamma$ is a future-directed null curve, but every two points on the curve can be joined by a timelike curve, given by the straight line $s\mapsto \gamma (t_1)+ s v$ $(s\in [0,1])$, where $t_1<t_2$ and $v:=\gamma (t_2)-\gamma (t_1)$ is future-directed timelike. This shows that $\gamma$ is timelike in the sense of Definition 2.18 and hence by Lemma 2.21 (iii) it is also a continuous timelike curve. This may be viewed as a caveat concerning the notion of continuous timelike curves as introduced in [57, p. 192f.].

#### Remark 2.23

The above considerations exemplify the fact that our notion of timelike curves corresponds to maps from intervals in $\mathbb {R}$ into *X* that preserve the chronal order, hence could be called *isochronal*, while null curves correspond to *achronal* curves. However, we feel that the danger of confusion is rather low and have therefore opted for the above, more intuitive, definitions.

We now introduce the *length* of a causal curve via the time separation function $\tau$.

#### Definition 2.24

Let $\gamma :[a,b]\rightarrow X$ be a future-directed causal curve, then we define its $\tau$*-length* by

2 $$\begin{aligned} L_\tau (\gamma ):=\inf \left\{ \sum _{i=0}^{N-1} \tau (\gamma (t_i),\gamma (t_{i+1})): N\in \mathbb {N},\ a=t_0<t_1<\cdots <t_N=b\right\} , \end{aligned}$$

and analogously if $\gamma$ is a past-directed causal curve.

#### Lemma 2.25

Let $\gamma :[a,b]\rightarrow X$ be a future-directed causal curve and $c\in (a,b)$. Then,

3 $$\begin{aligned} L_\tau (\gamma ) = L_\tau (\gamma |_{[a,c]}) + L_\tau (\gamma |_{[c,b]}). \end{aligned}$$

#### Proof

A partition of [*a*, *c*] and a partition of [*c*, *b*] give naturally a partition of [*a*, *b*], hence $L_\tau (\gamma ) \le L_\tau (\gamma |_{[a,c]}) + L_\tau (\gamma |_{[c,b]})$. On the other hand, given a partition $a=t_0<t_1<\cdots <t_N=b$ of [*a*, *b*], we have to consider two cases. The first case is when there is a $k\in \{1,\ldots ,N\}$ such that $t_k = c$. Consequently, $(t_i)_{i=0}^k$ is a partition of [*a*, *c*], and $(t_i)_{i=k}^N$ is a partition of [*c*, *b*]. Thus,

4 $$\begin{aligned} L_\tau (\gamma |_{[a,c]}) + L_\tau (\gamma |_{[c,b]})\le \sum _{i=0}^{N-1} \tau (\gamma (t_i),\gamma (t_{i+1})). \end{aligned}$$

If there is no such *k*, then define $j:=\max \{1\le i \le N: t_i< c\}$. Then, $(t_i)_{i=0}^j \cup \{c\}$ is a partition of [*a*, *c*] and $\{c\}\cup (t_i)_{i=j}^N$ is a partition of [*c*, *b*]. Hence,

$$\begin{aligned} L_\tau (\gamma |_{[a,c]}) + L_\tau (\gamma |_{[c,b]})&\le \sum _{i=0}^{j-1}\Big (\tau (\gamma (t_i),\gamma (t_{i+1}))\Big ) + \tau (\gamma (t_j),\gamma (c))\\&\quad + \tau (\gamma (c),\gamma (t_{j+1}))+ \sum _{i=j+1}^{N-1} \Big (\tau (\gamma (t_i),\gamma (t_{i+1}))\Big )\\&\le \sum _{i=0}^{N-1} \tau (\gamma (t_i),\gamma (t_{i+1})), \end{aligned}$$

where in the last inequality we used the reverse triangle inequality. Taking now the infimum over all partitions of [*a*, *b*], we obtain $L_\tau (\gamma |_{[a,c]}) + L_\tau (\gamma |_{[c,b]})\le L_\tau (\gamma )$. $\square$

#### Definition 2.26

By a *reparametrization* of a future-directed causal curve $\gamma :[a,b]\rightarrow X$, we mean a future-directed causal curve $\lambda :[c,d]\rightarrow X$ with $\gamma = \lambda \circ \phi$, where $\phi :[a,b]\rightarrow [c,d]$ is continuous and strictly monotonically increasing.

Note that implicit in this definition is the assumption that $\lambda \circ \phi$ is Lipschitz continuous and observe that the inverse of such a $\phi$ is also strictly monotonically increasing and continuous.

#### Lemma 2.27

A reparametrization does not change the causality, i.e., the causal character is the same (timelike, null, causal).

#### Proof

Let $\gamma = \lambda \circ \phi :[a,b]\rightarrow X$ be a (without loss of generality) future-directed causal (timelike or null) curve and its reparametrization given via $\phi :[a,b]\rightarrow [c,d]$. Then, $\lambda$ is causal (timelike or null). To see this, let $c\le s_1 < s_2 \le d$. Since $\phi ^{-1}$ is strictly monotonically increasing we have that $t_1:= \phi ^{-1}(s_1) < \phi ^{-1}(s_2)=:t_2$ and thus $\lambda (s_1) = \gamma (t_1)\le \gamma (t_2) = \lambda (s_2)$ ($\lambda (s_1) \ll \lambda (s_2)$ or ). $\square$

#### Lemma 2.28

The $\tau$-length is reparametrization invariant.

#### Proof

Let $\gamma :[a,b]\rightarrow X$ be a future-directed causal curve and $\lambda :[c,d]\rightarrow X$ a reparametrization of $\gamma$ given via $\phi :[a,b]\rightarrow [c,d]$ (i.e., $\gamma = \lambda \circ \phi$). Let $a=t_0<t_1<\cdots <t_N = b$ be a partition of [*a*, *b*]. This yields a partition $c = t_0'< t_1'<\cdots <t_N' = d$ via $t_i':=\phi (t_i)$. Consequently, we have

$$\begin{aligned} L_\tau (\lambda )&\le \sum _{i=0}^{N-1}\tau (\lambda (t_i'),\lambda (t_{i+1}')) = \sum _{i=0}^{N-1}\tau (\lambda (\phi (t_i)),\lambda (\phi (t_{i+1})))\\&= \sum _{i=0}^{N-1}\tau (\gamma (t_i),\gamma (t_{i+1})). \end{aligned}$$

Now taking the infimum over all partitions of [*a*, *b*] yields $L_\tau (\lambda )\le L_\tau (\gamma )$ and thus by symmetry also $L_\tau (\gamma )\le L_\tau (\lambda )$. $\square$

#### Definition 2.29

A future-directed causal curve $\gamma :[a,b]\rightarrow X$ is called *rectifiable* if $L_\tau (\gamma |_{[t_1,t_2]})>0$ for all $a\le t_1 < t_2\le b$.

#### Lemma 2.30

A rectifiable causal curve is timelike.

#### Proof

Let $\gamma :[a,b]\rightarrow X$ be a future-directed rectifiable causal curve. Then, for $a\le t_1<t_2\le b$ we have $0<L_\tau (\gamma |_{[t_1,t_2]}) \le \tau (\gamma (t_1),\gamma (t_2))$. Thus, $\gamma (t_1)\ll \gamma (t_2)$ and $\gamma$ is timelike. $\square$

#### Example 2.31

(*A timelike curve with* $\tau$-*length zero, hence non-rectifiable*) Let $\gamma :\mathbb {R}\rightarrow \mathbb {R}^3_1$, $\gamma (t)=(t,\cos (t),\sin (t))$ ($t\in \mathbb {R}$) be the timelike curve given in Example 2.22. Let $t_1<t_2$ and let $t_1=s_0< s_1<\cdots < s_k = t_2$ be a partition of $[t_1,t_2]$. Then $L_\tau (\gamma |_{[t_1,t_2]}) \le \sum _{i=0}^{k-1} \tau (\gamma (s_i),\gamma (s_{i+1})) = \sum _{i=0}^{k-1} (-\eta (v_i,v_i))$, where $v_i:={\gamma (s_{i+1})-\gamma (s_i)}$ and $\eta$ is the Minkowski metric. It is not hard to see that $0<-\eta (v_i,v_i) = (s_{i+1}-s_i)^2 - 2 (1-\cos (s_{i+1}-s_i)) \le \frac{(s_{i+1}-s_i)^4}{12} + \frac{2(s_{i+1}-s_i)^6}{6!}\rightarrow 0$, for $s_{i+1}-s_i\rightarrow 0$. Consequently, for $\varepsilon >0$ one can choose a partition of $[t_1,t_2]$ with mesh-size $\delta$ sufficiently small such that $\sum _{i=0}^{k-1}(-\eta (v_i,v_i))\le k (\frac{\delta ^4}{12} + \frac{2\delta ^6}{6!}) <\varepsilon$, which shows that $L_\tau (\gamma )=0$. This is a direct proof for this specific curve of the general fact that for smooth and strongly causal spacetimes the $\tau$-length agrees with the length given by the norm, which we establish in the following proposition.

#### Proposition 2.32

Let $(M,d^h,\ll ,\le ,\tau )$ be the Lorentzian pre-length space induced by the smooth spacetime (*M*, *g*), see Example 2.11. Moreover, denote by $L_g(\gamma )=\int _a^b{\sqrt{-g({\dot{\gamma }},{\dot{\gamma }})}}\,\mathrm{d}t$ the length of a causal curve $\gamma :[a,b]\rightarrow M$ with respect to *g*. If (*M*, *g*) is strongly causal, then $L_\tau (\gamma )=L_g(\gamma )$.

#### Proof

By Lemma 2.21 (i) we know that the notions of causal curves coincide. Let $\gamma :[a,b]\rightarrow M$ be a future-directed causal curve. Let $a=t_0<t_1<\cdots <t_N=b$ be a partition of [*a*, *b*], then

$$\begin{aligned} L_g(\gamma ) = \sum _{i=0}^{N-1} L_g(\gamma |_{[t_i,t_{i+1}]}) \le \sum _{i=0}^{N-1}\tau (\gamma (t_i),\gamma (t_{i+1})). \end{aligned}$$

Taking now the infimum over all partitions of [*a*, *b*], we obtain $L_g(\gamma )\le L_\tau (\gamma )$.

Let $t\in [a,b]$ such that ${\dot{\gamma }}(t)$ exists and is future-directed causal and set $e:=\mathrm {exp}_{\gamma (t)}:{\tilde{U}}\overset{\cong }{\longrightarrow } U$, where *U* is a convex neighborhood of $\gamma (t)$ such that *e* is a diffeomorphism from ${\tilde{U}}:=e^{-1}(U)$ onto *U*. Let $V\subseteq U$ be a neighborhood of $\gamma (t)$ such that every causal curve with endpoints in *V* is contained in *U* and let $\delta >0$ be such that $\gamma ([t,t+\delta ])\subseteq V$. Then, we obtain for $0<h<\delta$

$$\begin{aligned} \frac{1}{h} \tau (\gamma (t),\gamma (t+h)) = \Vert \frac{1}{h} e^{-1}(\gamma (t+h))\Vert , \end{aligned}$$

where $\Vert .\Vert = \sqrt{|g(.,.)|}$. Thus, taking the limit $h\searrow 0$ we get

5 $$\begin{aligned} \lim _{h\searrow 0} \frac{\tau (\gamma (t),\gamma (t+h))}{h} = \Big \Vert \frac{\mathrm{d}}{\mathrm{d} h}\Big |_0\, e^{-1}(\gamma (t+h)) \Big \Vert = \Vert (T_0 \mathrm {exp}_{\gamma (t)})^{-1}({\dot{\gamma }}(t))\Vert = \Vert {\dot{\gamma }}(t)\Vert ,\quad \end{aligned}$$

where we used that $T_0 \exp _{\gamma (t)} = \mathrm {id}$ and that $e^{-1}\circ \gamma$ is differentiable at *t*, and so the one-sided derivative agrees with the derivative. Furthermore,

$$\begin{aligned} \frac{\tau (\gamma (t),\gamma (t+h))}{h} \ge \frac{1}{h}L_\tau (\gamma |_{[t,t+h]}) \ge \frac{1}{h} L_g(\gamma |_{[t,t+h]}) = \frac{1}{h}\int _t^{t+h}\Vert {\dot{\gamma }}(s)\Vert \mathrm {d}s, \end{aligned}$$

where we used the above ($L_\tau \ge L$). Now the left hand side goes to $\Vert {\dot{\gamma }}(t)\Vert$ as $h\searrow 0$ by (5) and obviously so does the right hand side. Consequently, $\lim _{h\searrow 0}\frac{1}{h}L_\tau (\gamma |_{[t,t+h]})=\Vert {\dot{\gamma }}(t)\Vert$ as well. Finally, we obtain for any segment of $\gamma$ that is contained in such a convex neighborhood *U*, say $\gamma ([t_0,t_1])\subseteq U$ and almost all *t* in $[t_0,t_1]$ that

6 $$\begin{aligned} D_+(t\mapsto L_\tau (\gamma |_{[t_0,t]})) = \Vert {\dot{\gamma }}(t)\Vert , \end{aligned}$$

where $D_+$ denotes the right-sided derivative.

We now establish that $\phi :[t_0,t_0+\delta ]\rightarrow [0,\infty )$ given by $\phi (t):= L_\tau (\gamma |_{[t_0,t]})$ is absolutely continuous. Let $([a_i,b_i])_{i=1}^N$ be a collection of non-overlapping intervals in $[t_0,t_0+\delta ]$ with $\sum _{i=1}^N (b_i-a_i) < \alpha$, $\alpha$ to be given later. Then, we calculate

$$\begin{aligned} \sum _{i=1}^N |\phi (b_i)-\phi (a_i)| = \sum _{i=1}^N L_\tau (\gamma |_{[a_i,b_i]})\le \sum _{i=1}^N \tau (\gamma (a_i),\gamma (b_i))=:(*), \end{aligned}$$

where we used that $\phi$ is monotonically increasing (cf. Lemma 3.33) and that $L_\tau$ is additive by Lemma 2.25. In the convex neighborhood *U*, we know that for $p< q$ with $p,q \in V$, the maximal causal curve joining *p* and *q* is contained in *U* and its length is given by $\Vert \Delta (p,q)\Vert$. Here $\Delta :=E^{-1}:U\times U \rightarrow TM$ is a diffeomorphism onto its image and $E(v) = (\pi (v),\mathrm {exp}(v))$ for $v\in TM$ in the domain of *E*, cf. [41, Lemma 5.9]. This implies that

$$\begin{aligned} (*)\le \sum _{i=1}^N \Vert \Delta (\gamma (a_i),\gamma (b_i))\Vert \le C \sum _{i=1}^N \Vert \Delta (\gamma (a_i),\gamma (b_i))\Vert _2=:(**), \end{aligned}$$

for some constant *C* (depending only on *g* and *U*), where $\Vert .\Vert _2$ denotes the Euclidean norm in these coordinates. Since $\Delta (p,.)$ is smooth, it is locally Lipschitz continuous, and since $\Delta (p,p)=0$ for all $p\in U$ we get $\Vert \Delta (\gamma (a_i),\gamma (b_i))\Vert _2 = \Vert \Delta (\gamma (a_i),\gamma (b_i)) - \Delta (\gamma (a_i),\gamma (a_i))\Vert _2\le C' \Vert \gamma (a_i)-\gamma (b_i)\Vert _2$ $\le {\tilde{C}}(b_i-a_i)$. In the last inequality, we used the Lipschitz continuity of $\gamma$. Finally, we get for $\varepsilon >0$ and $0< \alpha <\frac{\varepsilon }{C {\tilde{C}}}$ that

$$\begin{aligned} (**)\le C {\tilde{C}} \sum _{i=1}^N (b_i-a_i)< C {\tilde{C}} \alpha < \varepsilon , \end{aligned}$$

establishing the absolute continuity of $\phi$. It follows that there exists a subset of full measure in $[t_0,t_0+\delta ]$ on which $\phi$ is differentiable and where its derivative is given by (6). This enables us to apply the Fundamental Theorem of Calculus to obtain

$$\begin{aligned} L_\tau (\gamma |_{[t_0,t_1]}) = \int _{t_0}^{t_1} \frac{\mathrm{d}}{\mathrm{d} s}L_\tau (\gamma |_{[t_0,s]})\mathrm {d}s = \int _{t_0}^{t_1} \Vert {\dot{\gamma }}(s)\Vert \mathrm {d}s = L_g(\gamma |_{[t_0,t_1]}). \end{aligned}$$

Covering $\gamma ([a,b])$ with finitely many such neighborhoods *V* and using the additivity of both $L_\tau$ (Lemma 2.25) and *L* yields $L_\tau (\gamma ) = L_g(\gamma )$. $\square$

### Maximal causal curves

#### Definition 2.33

Let $(X,d,\ll ,\le ,\tau )$ be a Lorentzian pre-length space. A future-directed causal curve $\gamma :[a,b]\rightarrow X$ is *maximal* if $L_\tau (\gamma ) = \tau (\gamma (a),\gamma (b))$, and analogously for past-directed causal curves.

A note on terminology is in order here: According to the above definition, a maximal curve $\gamma$ is a time separation realizing curve. Any such $\gamma$ is also maximal in the following sense: Let $\sigma$ be another causal curve connecting $p=\gamma (a)$ and $q=\gamma (b)$ with $L_\tau (\sigma )\ge L_\tau (\gamma )$. Then, $L_\tau (\sigma ) = L_\tau (\gamma )$: In fact, by the definition of $\tau$-length we have $L_\tau (\sigma )\le \tau (p,q)=L_\tau (\gamma )$. For Lorentzian length spaces (see Sect. 3) both notions of maximality in fact coincide.

#### Proposition 2.34

Let $(X,d,\ll ,\le ,\tau )$ be a Lorentzian pre-length space.

(i) A null curve is always maximal on any compact interval.
(ii) A maximal curve is maximal on any subinterval.
(iii) If a maximal curve is timelike then it is rectifiable.

#### Proof

Let $\gamma :[a,b]\rightarrow X$ be a future-directed causal curve.

(i) Let $\gamma$ be null. Then, for all $a\le t_1 < t_2 \le b$ we have $\tau (\gamma (t_1),\gamma (t_2))= 0$, which implies $L_\tau (\gamma ) = 0$. Thus, $L_\tau (\gamma ) = 0 = \tau (\gamma (a),\gamma (b))$ and $\gamma$ is maximal.
(ii) Let $\gamma$ be maximal and $a\le c < d \le b$ a subinterval. Assume that $\gamma$ is not maximal on [*c*, *d*], i.e., $L_\tau (\gamma |_{[c,d]}) < \tau (\gamma (c),\gamma (d))$. Then by Lemma 2.25 and the reverse triangle inequality, we get
  $$\begin{aligned} \tau (\gamma (a),\gamma (b))&= L_\tau (\gamma ) = L_\tau (\gamma |_{[a,c]}) + L_\tau (\gamma |_{[c,d]}) + L_\tau (\gamma |_{[d,b]})\\&< L_\tau (\gamma |_{[a,c]}) +\tau (\gamma (c),\gamma (d)) + L_\tau (\gamma |_{[d,b]})\\&\le \tau (\gamma (a),\gamma (c)) + \tau (\gamma (c),\gamma (d)) + \tau (\gamma (d),\gamma (b))\\&\le \tau (\gamma (a),\gamma (b)). \end{aligned}$$
  This is a contradiction, thus establishing that $\gamma$ is maximal on [*c*, *d*].
(iii) Let $\gamma$ be timelike. Then, for all $a\le t_1 < t_2 \le b$ we have $0<\tau (\gamma (t_1),\gamma (t_2))= L_\tau (\gamma |_{[t_1,t_2]})$. Thus, $\gamma$ is rectifiable.

$$\square $$

### Causality conditions

#### Definition 2.35

A causal space $(X,\ll ,\le )$ is called

(i) *chronological* if the relation $\ll$ is irreflexive, i.e.,  for all $x\in X$, and
(ii) *causal* if the relation $\le$ is a partial order, i.e., $x\le y$ and $y\le x$ implies that $x=y$ for all $x,y\in X$.
  A Lorentzian pre-length space $(X,d,\ll ,\le ,\tau )$ is called
(iii) *non-totally imprisoning* if for every compact set $K\Subset X$ there is a $C>0$ such that the *d*-arclength of all causal curves contained in *K* is bounded by *C*,
(iv) *strongly causal* if the Alexandrov topology ${\mathcal {A}}$ agrees with the metric topology ${\mathcal {D}}$ (and hence also with the chronological topology ${\mathcal {I}}$), and
(v) *globally hyperbolic* if $(X,d,\ll ,\le ,\tau )$ is non-totally imprisoning and for every $x,y\in X$ the set $J^+(x)\cap J^-(y)$ is compact in *X*.

#### Remark 2.36

Causality does not imply chronology in general as can be seen from the simple example: $X:=\{*\}$, $\ll :=\le := \{(*,*)\}$. Clearly, both $\ll$ and $\le$ are transitive and reflexive; hence, $(X,\ll ,\le )$ is not chronological, but it is causal.

#### Definition 2.37

A causal space $(X,\ll ,\le )$ is called *interpolative* if for all $x,y\in X$ with $x\ll y$ there is a $z\in X$ such that $x\ll z\ll y$ and $x\ne z\ne y$.

#### Lemma 2.38

Let $(X,d,\ll ,\le ,\tau )$ be a Lorentzian pre-length space. Then,

(i) if $(X,d,\ll ,\le ,\tau )$ is causal and interpolative it is chronological,
(ii) if $(X,d,\ll ,\le ,\tau )$ is chronological then the time separation function $\tau$ is zero on the diagonal, i.e., $\tau (x,x)=0$ for all $x\in X$, and
(iii) if $(X,d,\ll ,\le ,\tau )$ is strongly causal, then for all $x\in X$, for every neighborhood *U* of *x*, there is a neighborhood $V\subseteq U$ of *x* such that for every causal curve $\gamma :[a,b]\rightarrow X$ with $\gamma (a),\gamma (b)\in V$ one has $\gamma ([a,b])\subseteq U$ (i.e., the usual definition of strong causality for spacetimes).

#### Proof

(i) Assume that there is an $x\in X$ such that $x\ll x$. Then, since $(X,d,\ll ,\le ,\tau )$ is interpolative there is a $z\in X$ such that $x\ll z \ll x$ and $x\ne z$. This implies $x\le z \le x$ and since $\le$ is a partial order $x=z$—a contradiction.
(ii) This follows from $\tau (x,x)>0 \Leftrightarrow x\ll x$ for all $x\in X$.
(iii) Let $x\in X$ and let *U* be a neighborhood of *x*. Then, since ${\mathcal {A}}={\mathcal {D}}$ there is a $V\in {\mathcal {A}}$ such that $x\in V$ and $V\subseteq U$. We may assume that $V=(I^+(x_1)\cap I^-(y_1)) \cap \cdots \cap (I^+(x_n)\cap I^-(y_n))$ for some $x_1,y_1,\ldots ,x_n,y_n\in X$. Now let $\gamma :[a,b]\rightarrow X$ be a (without loss of generality) future-directed causal curve with $\gamma (a),\gamma (b)\in V$. We claim that $\Gamma \subseteq V$, thus $\gamma ([a,b])\subseteq U$. Let $t\in [a,b]$, then $\gamma (t)\in J^+(\gamma (a))$. For each $1\le i \le n$, we have $x_i\ll \gamma (a)\le \gamma (t)$ and so $x_i\ll \gamma (t)$ by push-up (Lemma 2.10). This is equivalent to $\gamma (t)\in I^+(x_i)$. Analogously, one shows that $\gamma (t)\in I^-(y_i)$.

$$\square $$

It is not clear at this moment if strong causality is equivalent to the condition of Lemma 2.38 (iii), i.e., the nonexistence of almost closed causal curves, as is the case for smooth spacetimes, see [40, Thm. 3.27] without further assumptions or more structure on the Lorentzian pre-length space. However, for Lorentzian length spaces the crucial additional ingredient will be *localizability* and for these spaces the conditions will be equivalent, see Theorem 3.26 (iv).

## Lorentzian length spaces

### Causal connectedness

#### Definition 3.1

A Lorentzian pre-length space $(X,d,\ll ,\le ,\tau )$ is called *causally path connected* if for all $x,y\in X$ with $x\ll y$ there is a future-directed timelike curve from *x* to *y* and for $x<y$ there is a future-directed causal curve from *x* to *y*.

#### Lemma 3.2

A causally path connected Lorentzian pre-length space is interpolative.

#### Proof

Let $x,y\in X$ with $x\ll y$, then there is a future-directed timelike curve $\gamma :[a,b]\rightarrow X$ from *x* to *y*. Since $\gamma$ is not constant, there is a $t\in [a,b]$ with $x=\gamma (a)\ne \gamma (t)=:z$, and because $\gamma$ is timelike we have $x\ll z$. If $x=y$ we are done, and if $x\ne y$ there is a $\delta >0$ such that ${\bar{B}}^d_\delta (x)\cap {\bar{B}}^d_\delta (y)=\emptyset$. Then, if $\gamma ([a,b])\subseteq {\bar{B}}^d_\delta (x)\cup {\bar{B}}^d_\delta (y)$ it would follow that $\gamma ([a,b])$ can be written as a disjoint union of the non-empty closed sets ${\bar{B}}^d_\delta (x)\cap \gamma ([a,b])$ and ${\bar{B}}^d_\delta (y)\cap \gamma ([a,b])$, contradicting connectedness. Thus, there is a $t'\in [a,b]$ such that $x\ne z=\gamma (t') \ne y$ and by assumption $x\ll z \ll y$. $\square$

#### Lemma 3.3

Let $(X,d,\ll ,\le ,\tau )$ be a causally path connected Lorentzian pre-length space.

(i) $(X,d,\ll ,\le ,\tau )$ is chronological if and only if there are no closed timelike curves in *X*.
(ii) $(X,d,\ll ,\le ,\tau )$ is causal if and only if there are no closed causal curves in *X*.

#### Proof

Let $(X,d,\ll ,\le ,\tau )$ be a causally path connected Lorentzian pre-length space, which for brevity we just denote by *X*.

(i)
  ($\Rightarrow$): Let *X* be chronological and $\gamma$ a closed timelike curve. Then, for all *x* in the image of $\gamma$ we have $x\ll x$—a contradiction.
  ($\Leftarrow$): Let *X* be such that there are no closed timelike curves. Let $x\in X$ with $x\ll x$, then by the causal path-connectedness there is a future-directed timelike curve from *x* to *x*—a contradiction.
(ii)
  ($\Rightarrow$): Let *X* be causal and $\gamma$ a closed causal curve. Since $\gamma$ is not constant, there are points *x*, *y* on $\gamma$ with $x\ne y$ and by assumption $x\le y\le x$—a contradiction.
  ($\Leftarrow$): Let *X* be such that there are no closed causal curves. Let $x,y\in X$ with $x< y< x$, then by the causal path-connectedness there is a future-directed causal curve from *x* to *y* to *x*—a contradiction.

$$\square $$

### Limit curves

#### Definition 3.4

Let $(X,d,\ll ,\le ,\tau )$ be a Lorentzian pre-length space and let $x\in X$. A neighborhood *U* of *x* is called *causally closed* if $\le$ is closed in ${\bar{U}}\times {\bar{U}}$, i.e., if $p_n,q_n\in U$ with $p_n\le q_n$ for all $n\in \mathbb {N}$ and $p_n\rightarrow p\in {\bar{U}}$, $q_n\rightarrow q\in {\bar{U}}$, then $p\le q$. A Lorentzian pre-length space $(X,d,\ll ,\le ,\tau )$ is called *locally causally closed* if every point has a causally closed neighborhood.

#### Proposition 3.5

Strongly causal spacetimes with continuous metrics are locally causally closed.

#### Proof

Let (*M*, *g*) be a strongly causal spacetime with *g* continuous. Then, (*M*, *g*) is non-totally imprisoning, cf. [48, p. 1437]. Let $p\in M$ and *U* an open, relatively compact neighborhood of *p*, then by [48, Lemma 2.7] there is a $C>0$ such that $L^h(\gamma )\le C$ for all causal curves $\gamma$ with image contained in *U*, where *h* is a complete Riemannian metric on *M*. Strong causality implies the existence of a neighborhood *V* of *p*, $V\subseteq U$ such that for all causal curves $\lambda :[a,b]\rightarrow M$ with $\lambda (a),\lambda (b)\in V$ one has $\lambda ([a,b])\subseteq U$. Now let $(x_n)_n$, $(y_n)_n$ be sequences in *V* with $x_n\le y_n$ for all $n\in \mathbb {N}$ and $x_n\rightarrow x\in {\bar{V}}$, $y_n\rightarrow y\in {\bar{V}}$. Thus, there is a sequence $(\gamma _n)_n$ of future-directed causal curves $\gamma _n:[0,1]\rightarrow M$ with $\gamma _n(0)=x_n\in V$, $\gamma _n(1)=y_n\in V$ for all $n\in \mathbb {N}$. Hence $\gamma _n([0,1])\subseteq U$ and so $L^h(\gamma _n)\le C$ for all $n\in \mathbb {N}$. Finally, the limit curve theorem [48, Thm. 1.5] establishes the existence of a future-directed causal curve from *x* to *y*, if $x\not =y$, thus $x<y$. $\square$

#### Lemma 3.6

Let $(X,d,\ll ,\le ,\tau )$ be a locally causally closed Lorentzian pre-length space and let $(\gamma _n)_n$ be a sequence of future-directed causal curves $\gamma _n:[a,b]\rightarrow X$ converging pointwise to a non-constant Lipschitz curve $\gamma :[a,b]$ $\rightarrow X$. Then, $\gamma$ is a future-directed causal curve.

#### Proof

For every $t\in [a,b]$, there is an open, causally closed neighborhood $U_t$ of $\gamma (t)$. Let $a\le t_1<t_2\le b$ such that $\gamma (t_1),\gamma (t_2)\in U_t$. Then, there is an $n_0\in \mathbb {N}$ such that for all $n\ge n_0$ we have $\gamma _n(t_1),\gamma _n(t_2)\in U_t$. Since $\gamma _n(t_1)\le \gamma _n(t_2)$ for all $n\in \mathbb {N}$ and by assumption $\gamma _n(t_i)\rightarrow \gamma (t_i)$ for $i=1,2$, we conclude that $\gamma (t_1)\le \gamma (t_2)$. This gives an open cover of the compact set $\gamma ([a,b])$, from which we may extract a finite sub-cover $U_1,\ldots ,U_N$. Additionally, this finite cover has a Lebesgue number $\delta >0$. Let $L>0$ be the Lipschitz constant of $\gamma$, then if $|t_1-t_2|\le \frac{\delta }{L}$, one has $\gamma (t_1),\gamma (t_2)\in U_i$ for some $i\in \{1,\ldots ,N\}$. Now let $a\le t_1<t_2\le b$ and let $t_1 =: s_0< s_1<\cdots< s_{k-1} < s_k:= t_2$ such that $|s_{j+1}-s_j|\le \frac{\delta }{L}$ for all $j\in \{0,\ldots ,k-1\}$. Thus, by construction $\gamma (s_j),\gamma (s_{j+1})\in U_{l_j}$ for all $j\in \{0,\ldots ,k-1\}$ and corresponding $l_j\in \{1,\ldots ,N\}$ and so $\gamma (t_1) = \gamma (s_0)\le \gamma (s_1)\le \cdots \le \gamma (s_k)=\gamma (t_2)$, hence $\gamma (t_1)\le \gamma (t_2)$. $\square$

#### Theorem 3.7

(Limit curve theorem) Let $(X,d,\ll ,\le ,\tau )$ be a locally causally closed Lorentzian pre-length space. Let $(\gamma _n)_n$ be a sequence of future-directed causal curves $\gamma _n:[a,b]\rightarrow X$ that are uniformly Lipschitz continuous, i.e., there is an $L>0$ such that $\mathrm {Lip}(\gamma _n)\le L$ for all $n\in \mathbb {N}$. Suppose that there exists a compact set that contains every $\gamma _n([a,b])$ or that *d* is proper (i.e., all closed and bounded sets are compact) and that the curves $(\gamma _n)_n$ accumulate at some point, i.e., there is a $t_0\in [a,b]$ such that $\gamma _n(t_0)\rightarrow x_0\in X$. Then, there exists a subsequence $(\gamma _{n_k})_k$ of $(\gamma _n)_n$ and a Lipschitz continuous curve $\gamma :[a,b]\rightarrow X$ such that $\gamma _{n_k}\rightarrow \gamma$ uniformly. If $\gamma$ is non-constant, then $\gamma$ is a future-directed causal curve. In particular, if $\gamma _n(a)=p$, $\gamma _n(b)=q$ for all $n\in \mathbb {N}$, with $p\ne q$, then $\gamma$ is a future-directed causal curve connecting *p* and *q*.

#### Proof

The sequence $(\gamma _n)_n$ is equicontinuous and either the $\gamma _n$s are contained in a compact set by assumption or (*X*, *d*) is proper. In the latter case, we have for $t\in [a,b]$ and all $n\in \mathbb {N}$

$$\begin{aligned} d(x_0,\gamma _n(t))&\le d(x_0,\gamma _n(t_0)) + d(\gamma _n(t_0),\gamma _n(t))\\&\le C + \mathrm {Lip}(\gamma _n)|t-t_0| \le C + L (b-a), \end{aligned}$$

where $C>0$ is some constant determined by the convergence of $\gamma _n(t_0)$ to $x_0$. Thus, in both cases $(\gamma _n(t))_n$ is relatively compact for all $t\in [a,b]$ and so we can apply the Arzelà-Ascoli theorem (e.g., [42, Thm. 1.4.9]) to get a uniformly converging subsequence $(\gamma _{n_k})_k$. The uniform limit $\gamma :=\lim _{k\rightarrow \infty }\gamma _{n_k}$ is Lipschitz continuous, with $\mathrm {Lip}(\gamma )\le L$, and thus, Lemma 3.6 shows that $\gamma$ is a future-directed causal curve. $\square$

#### Lemma 3.8

(A sufficient condition that the limit curve is not constant, cf. [35, Thm. 3.1]) Let $(\gamma _n)_n$ be a sequence of (continuous) curves defined on [*a*, *b*] that converge uniformly to a curve $\gamma :[a,b]\rightarrow X$. If there is a $t\in [a,b]$ and a neighborhood *U* of $\gamma (t)$ such that only finitely many $\gamma _n$ are contained in *U*, then $\gamma$ is not constant.

#### Proof

Let $\varepsilon >0$ be such that $B^d_\varepsilon (\gamma (t))\subseteq U$. The assumptions yield that there is an $n_0\in \mathbb {N}$ such that for all $n\ge n_0$ there is an $s_n\in [a,b]$ with $\gamma _n(s_n)\not \in U$, hence $0<\varepsilon \le d(\gamma (t),\gamma _n(s_n))$. Without loss of generality, we can assume that $s_n\rightarrow s^*\in [a,b]$, and thus, $0<\varepsilon \le d(\gamma (t),\gamma (s^*))$. $\square$

#### Remark 3.9

We do not require (*X*, *d*) to be a proper metric space in the definition of a Lorentzian pre-length space, since in the case where we will apply such results (as the Limit curve theorem) in the development of the theory, it will be to (relatively) compact subsets.

We now introduce inextendible causal curves.

#### Definition 3.10

Let $-\infty \le a < b\le \infty$ and let $\gamma :[a,b)\rightarrow X$ be a future (or past)-directed causal (or timelike) curve. It is called *extendible* if there exists a future (past)-directed causal (timelike) curve ${\tilde{\gamma }}:[a,b]\rightarrow X$ such that ${\tilde{\gamma }}|_{[a,b)}=\gamma$. The curve $\gamma$ is called *inextendible* if it is not extendible. Analogously for the other endpoint of the interval.

#### Remark 3.11

An extendible causal curve is Lipschitz continuous on its (open) domain of definition.

#### Lemma 3.12

Let $(X,d,\ll ,\le ,\tau )$ be a locally causally closed Lorentzian pre-length space; let $-\infty< a<b\le \infty$ and let $\gamma :[a,b)\rightarrow X$ be a (without loss of generality) future-directed causal curve parametrized with respect to *d*-arclength. If (*X*, *d*) is a proper metric space or the image of $\gamma$ is contained in a compact set, then $\gamma$ is inextendible if and only if $b=\infty$. In this case, $L^d(\gamma )=\infty$. Moreover, $\gamma$ is inextendible if and only if $\lim _{t\nearrow b}\gamma (t)$ does not exist.

#### Proof

We first show the equivalence of $\gamma$ inextendible and $b=\infty$.

($$\Leftarrow $$):
Since $\gamma$ is parametrized with respect to *d*-arclength, we have $d(\gamma (a),\gamma (t)) = t-a$ for all $t\in [a,b)$. Thus, if $\gamma$ were extendible we would have $b=d(\gamma (a),\gamma (b)) + a<\infty$—a contradiction.
($$\Rightarrow $$):
Assume that $b<\infty$. In both cases, we have that $\gamma$ is contained in a compact set. Either by assumption or if (*X*, *d*) is proper, then $\gamma ([a,b))\subseteq B^d_{b-a}(\gamma (a))$, which is relatively compact. Thus, there exists a sequence $(t_n)_n$ with $t_n\nearrow b$ and $\lim _{n\rightarrow \infty }\gamma (t_i)=:p$. This is the only limit point of $\gamma$ as the parameter approaches *b*. Assume that there is another sequence $(s_n)_n$ such that $s_n\nearrow b$ and $\lim _{n\rightarrow \infty }\gamma (s_n)=:q\ne p$. Then, we have $\delta := d(p,q)>0$, and thus, there is an $n_0\in \mathbb {N}$ such that for all $n\ge n_0$ we have $(b-t_n)<\frac{\delta }{4}$, $(b-s_n)<\frac{\delta }{4}$, $d(p,\gamma (t_n))<\frac{\delta }{4}$ and $d(q,\gamma (s_n))<\frac{\delta }{4}$. The curve $\gamma$ is 1-Lipschitz continuous, and so we obtain
  $$\begin{aligned} \delta&= d(p,q) \le d(p,\gamma (t_n)) + d(\gamma (t_n),\gamma (s_n)) + d(\gamma (s_n),q)\\&< \frac{\delta }{4} + |t_n - s_n| + \frac{\delta }{4} \le \frac{\delta }{2} + (b - t_n) + (b - s_n) < \delta , \end{aligned}$$
  a contradiction. At this point, we show that we can extend $\gamma$ via ${\tilde{\gamma }}$ given by ${\tilde{\gamma }}|_{[a,b)}:=\gamma$, ${\tilde{\gamma }}(b):=p$. Clearly, ${\tilde{\gamma }}$ is 1-Lipschitz continuous, and so it remains to show that ${\tilde{\gamma }}(t)\le p$ for all $t\in [a,b)$. Let *U* be a causally closed neighborhood of *p*. Then, there is a $t^*\in [a,b)$ such that for all $t\in (t^*,b)$, ${\tilde{\gamma }}(t)=\gamma (t)\in U$. Fix $t\in (t^*,b)$ and let $(t_n)_n$ be a sequence in (*a*, *b*) with $t_n\nearrow b$ and $t\le t_0$. This yields that $\gamma (t)\le \gamma (t_n)$ for all $n\in \mathbb {N}$ since $\gamma$ is causal. By construction $\gamma (t_n)\rightarrow p$ and hence by causal closedness of *U*, we obtain $\gamma (t)\le p$. This shows that $\gamma (t)\le p$ for all $t\in (t^*,b)$. Now fix $t\in [a,t^*]$ and let $t'\in (t^*,b)$. Then, $\gamma (t)\le \gamma (t')\le p$ and by transitivity $\gamma (t)\le p$, as required.

The latter implication shows that if $\lim _{t\nearrow b}\gamma (t)$ exists then $\gamma$ is extendible. Conversely, if $\gamma$ is extendible the limit obviously exists. $\square$

#### Definition 3.13

A Lorentzian pre-length space $(X,d,\ll ,\le ,\tau )$ is called *d-compatible* if for every $x\in X$ there exists a neighborhood *U* of *x* and a constant $C>0$ such that $L^d(\gamma )\le C$ for all causal curves $\gamma$ contained in *U*.

#### Theorem 3.14

(Limit curve theorem for inextendible curves) Let $(X,d,\ll ,\le ,\tau )$ be a locally causally closed and *d*-compatible Lorentzian pre-length space. Let $(\gamma _n)_n$ be a sequence of future-directed causal curves $\gamma _n:[0,L_n]\rightarrow X$ that are parametrized with respect to *d*-arclength with $L_n:=L^d(\gamma _n)\rightarrow \infty$. If there exists a compact set that contains every $\gamma _n([0,L_n])$ or if *d* is proper and $\gamma _n(0)\rightarrow x$ for some $x\in X$, then there exists a subsequence $(\gamma _{n_k})_k$ of $(\gamma _n)_n$ and a future-directed causal curve $\gamma :[0,\infty )\rightarrow X$ such that $\gamma _{n_k}\rightarrow \gamma$ locally uniformly. Moreover, $\gamma$ is inextendible.

#### Proof

Extend each $\gamma _n$ constantly to $[0,\infty )$ and denote it again by $\gamma _n$. Then, the sequence $(\gamma _n)_n$ is equicontinuous since every $\gamma _n$ is 1-Lipschitz continuous and as in the proof of Theorem 3.7 we have $\gamma _n([0,\infty ))\subseteq K$, for some compact set $K\Subset X$. Again, the Arzelà-Ascoli theorem gives a locally uniformly converging subsequence $(\gamma _{n_k})_k$. The limit curve $\gamma :=\lim _{k\rightarrow \infty }\gamma _{n_k}:[0,\infty )\rightarrow X$ is 1-Lipschitz continuous. To see that $\gamma$ is causal, observe that for every $t>0$ there is a $k_0\in \mathbb {N}$ such that $L_{n_k}\ge t$ for all $k\ge k_0$ and we have $\gamma _{n_k}|_{[0,t]}\rightarrow \gamma |_{[0,t]}$ uniformly. To apply Lemma 3.6, we need to show that $\gamma |_{[0,t]}$ is not constant (at least for $t>0$ sufficiently large). Let *U* be a neighborhood of $\gamma (0)$ such that there is a $C>0$ that bounds the *d*-arclength of all causal curves in *U* and let $t>C$. Then, since $L_{n_k}\ge t>C$, the $\gamma _{n_k}$s cannot be contained in *U*; hence, Lemma 3.8 yields that $\gamma |_{[0,t]}$ is not constant. Thus, $\gamma |_{[0,t]}$ is future-directed causal by Lemma 3.6, since $\gamma _{n_k}|_{[0,t]}$ is causal as a segment of the original causal curve $\gamma _{n_k}$ defined on $[0,L_{n_k}]$ for $k\ge k_0$. Now let $0\le t_1< t_2<\infty$, then, by the above, there is a $t\ge t_2$ such that $\gamma |_{[0,t]}$ is causal and hence $\gamma (t_1)\le \gamma (t_2)$. It remains to show the inextendibility of $\gamma$. Assume, to the contrary, that $\gamma$ is extendible and set $\lim _{t\nearrow \infty }\gamma (t)=:p$. Let *V* be a neighborhood of *p* such that there is a $C>0$ that bounds the *d*-arclength of all causal curves in *V*. There is a $t^*\in [0,\infty )$ such that $\gamma ([t^*,\infty ))\subseteq V$ and hence for $T>t^*$ with $T-t^*>C$ there is a $k\in \mathbb {N}$ such that $\gamma _{n_k}([t^*,T])\subseteq V$ by the uniform convergence on $[t^*,T]$ and $L_{n_k}>T$. This is a contradiction as $L^d(\gamma _{n_k}|_{[t^*,T]}) = T-t^* >C$. $\square$

#### Corollary 3.15

Let $(X,d,\ll ,\le ,\tau )$ be a locally causally closed and *d*-compatible Lorentzian pre-length space. Then, *X* is non-totally imprisoning if and only if no compact set in *X* contains an inextendible causal curve.

#### Proof

- ($\Rightarrow$): Assume that there is a compact set $K\Subset X$ and an inextendible causal curve $\gamma$ contained in *K*. By Lemma 3.12 we have $L^d(\gamma )=\infty$—a contradiction to *X* being non-totally imprisoning.
- ($\Leftarrow$): Assume that there is a compact set $K\Subset X$ and a sequence of (without loss of generality) future-directed causal curves $\gamma _n:I_n\rightarrow X$ contained in *K* with $L^d(\gamma _n)\rightarrow \infty$. Parametrizing them with respect to *d*-arclength gives a sequence $\lambda _n:[0,L_n]\rightarrow X$, with $L_n:=L^d(\gamma _n)=L^d(\lambda _n)$. Now Theorem 3.14 yields a limit curve of this sequence that is an inextendible causal curve contained in *K*—a contradiction.

$$\square $$

### Localizability

We now try to capture the idea that locally the geometry and causality of a (smooth) Lorentzian manifold is better behaved than globally. The following definition generalizes to our current setting a number of essential properties inherent to convex neighborhoods in smooth Lorentzian manifolds. Also in metric length spaces, the corresponding notion would be that of a convex neighborhood (in the sense of [3, Def. 3.6.5]).

#### Definition 3.16

A Lorentzian pre-length space $(X,d,\ll ,\le ,\tau )$ is called *localizable* if $\forall x\in X$ there is an open neighborhood $\Omega _x$ of *x* in *X* with the following properties:

(i) There is a $C>0$ such that $L^d(\gamma )\le C$ for all causal curves $\gamma$ contained in $\Omega _x$ (hence *X* is *d*-compatible).
(ii) There is a continuous map $\omega _x:\Omega _x \times \Omega _x\rightarrow [0,\infty )$ such that $(\Omega _x, d|_{\Omega _x\times \Omega _x}, \ll |_{\Omega _x\times \Omega _x}, \le |_{\Omega _x\times \Omega _x}, \omega _x)$ is a Lorentzian pre-length space with the following non-triviality condition: For every $y\in \Omega _x$ we have $I^\pm (y)\cap \Omega _x\ne \emptyset$.
(iii) For all $p,q\in \Omega _x$ with $p<q$ there is a future-directed causal curve $\gamma _{p,q}$ from *p* to *q* that is maximal in $\Omega _x$ and satisfies
  7 $$\begin{aligned} L_\tau (\gamma _{p,q}) = \omega _x(p,q) \le \tau (p,q). \end{aligned}$$
  (That the curve $\gamma _{p,q}$ is maximal in $\Omega _x$ means that for every other future-directed causal curve $\lambda$ connecting *p* and *q* with image contained in $\Omega _x$ we have that $L_\tau (\gamma _{p,q}) \ge L_\tau (\lambda )$.)

We call such a neighborhood $\Omega _x$ a *localizing neighborhood of* *x*. If, in addition, the neighborhoods $\Omega _x$ can be chosen such that

(iv) Whenever $p,q\in \Omega _x$ satisfy $p\ll q$ then $\gamma _{p,q}$ is timelike and strictly longer than any future-directed causal curve in $\Omega _x$ from *p* to *q* that contains a null segment,

then $(X,d,\ll ,\le ,\tau )$ is called *regularly localizable*. Finally, if every point $x\in X$ has a neighborhood basis of open sets $\Omega _x$ satisfying (i)–(iii), respectively (i)–(iv), then $(X,d,\ll ,\le ,\tau )$ is called *strongly localizable*, respectively *SR-localizable*.

#### Proposition 3.17

Let $(X,d,\ll ,\le ,\tau )$ be a strongly causal and localizable Lorentzian pre-length space. Then, $L_\tau$ is upper semicontinuous, i.e., if $(\gamma _n)_n$ is a sequence of future-directed causal curves (defined on [*a*, *b*]) converging uniformly to a future-directed causal curve $\gamma :[a,b]\rightarrow X$, then

8 $$\begin{aligned} L_\tau (\gamma )\ge \limsup _n L_\tau (\gamma _n). \end{aligned}$$

#### Proof

By strong causality (and Lemma 2.38 (iii)) every point $x\in X$ has an open neighborhood $U_x\subseteq \Omega _x$ such that any causal curve with endpoints in $U_x$ is contained in $\Omega _x$.

Let $\varepsilon >0$, then there is a partition $(t_i)_{i=0}^N$ of [*a*, *b*] such that

9 $$\begin{aligned} \sum _{i=0}^{N-1} \tau (\gamma (t_i),\gamma (t_{i+1})) < L_\tau (\gamma ) + \frac{\varepsilon }{2}. \end{aligned}$$

By making the partition finer (and by the reverse triangle inequality) we can assume that $\gamma (t_i),\gamma (t_{i+1})\in U_{x_i}\subseteq \Omega _{x_i}$ for some $x_i\in \gamma ([a,b])$, $i=0,\ldots ,N-1$.

Thus,

10 $$\begin{aligned} \sum _{i=0}^{N-1} \tau (\gamma (t_i),\gamma (t_{i+1})) \ge \sum _{i=0}^{N-1} \omega _{x_i}(\gamma (t_i),\gamma (t_{i+1})) =: \Delta . \end{aligned}$$

Now we choose $n_0\in \mathbb {N}$ such that for all $n\ge n_0$ we have $\gamma _n(t_i),\gamma _n(t_{i+1})\in U_{x_i}$ and $|\omega _{x_i}(\gamma (t_i),\gamma (t_{i+1})) - \omega _{x_i}(\gamma _n(t_i),\gamma _n(t_{i+1}))|< \frac{\varepsilon }{2 N}$ for $i=0,\ldots ,N-1$. By construction $\gamma _n([t_i,t_{i+1}]) \subseteq \Omega _{x_i}$. Localizability then implies that for every $i=0,\ldots ,N-1$ there is a future-directed causal curve $\lambda _i$ from $\gamma _n(t_i)$ to $\gamma _n(t_{i+1})$ that is maximal in $\Omega _{x_i}$ with $\omega _{x_i}(\gamma _n(t_i),\gamma _n(t_{i+1})) = L_\tau (\lambda _i)$ and so

$$\begin{aligned} \Delta&\ge \sum _{i=0}^{N-1} \omega _{x_i}(\gamma _n(t_i),\gamma _n(t_{i+1})) - \frac{\varepsilon }{2} = \sum _{i=0}^{N-1} L_\tau (\lambda _i) - \frac{\varepsilon }{2}\\&\ge \sum _{i=0}^{N-1} L_\tau (\gamma _n|_{[t_i,t_{i+1}]}) - \frac{\varepsilon }{2} = L_\tau (\gamma _n) - \frac{\varepsilon }{2}, \end{aligned}$$

where in the last step we used the additivity of the $\tau$-length proved in Lemma 2.25. Together with (10) this yields $L_\tau (\gamma )\ge L_\tau (\gamma _n) - \varepsilon$ for every $n\ge n_0$. $\square$

#### Theorem 3.18

In a regularly localizable Lorentzian pre-length space, maximal causal curves have a causal character, i.e., if for a (future-directed) maximal causal curve $\gamma :[a,b]\rightarrow X$ there are $a\le t_1 < t_2\le b$ with $\gamma (t_1)\ll \gamma (t_2)$, then $\gamma$ is timelike. Otherwise, it is null.

#### Proof

First we establish that it suffices to show the claim for $t_1 = a$ and $t_2=b$. Indeed, let $\gamma :[a,b]\rightarrow X$ be a future-directed maximal causal curve and assume that there are $a<t_1<t_2<b$ with $x:=\gamma (t_1)\ll \gamma (t_2)=:y$. Thus, $\gamma (a)\le x\ll y \le \gamma (b)$ and hence by push-up (Lemma 2.10) we conclude that $\gamma (a)\ll \gamma (b)$.

We begin the main part of the proof by showing that the claim follows if there exist points $\gamma (t_1)=x\ll y=\gamma (t_2)$ such that $\gamma ([t_1,t_2])$ lies in a regularly localizing neighborhood $\Omega$ as in Definition 3.16. In fact, since $\gamma$ is maximizing on [*a*, *b*], it also is on $[t_1,t_2]$ (Proposition 2.34 (ii)), and we claim that $\gamma |_{[t_1,t_2]}$ is timelike. Otherwise, there would exist $t_1\le s_1 < s_2\le t_2$ such that $r_1:=\gamma (s_1) \not \ll \gamma (s_2)=:r_2$, implying that $\tau (r_1,r_2)=0$ and thus by maximality $L_\tau (\gamma |_{[s_1,s_2]})=0$, i.e., $\gamma |_{[s_1,s_2]}$ is null. By regular localizability (in $\Omega$) we know that there is a future-directed timelike curve $\gamma _{x,y}$ in $\Omega$ from *x* to *y* that is strictly longer than $\gamma |_{[t_1,t_2]}$, since the latter contains the null segment $\gamma |_{[s_1,s_2]}$. This is a contradiction to the maximality of $\gamma$. We now cover $\gamma ([t_1,b])$ by finitely many regularly localizing neighborhoods $\Omega _1=\Omega , \dots , \Omega _N$ as in Definition 3.16 and pick $t_i$ ($i=3,\dots ,N+2$), $t_{N+2}=b$, such that $\gamma (t_i) \in \Omega _{i-2}\cap \Omega _{i-1}$ for $i=3,\dots ,N+1$ and $\gamma ([t_i,t_{i+1}])\subseteq \Omega _{i-1}$ for $i=2,\dots N+1$. It then follows as above that, since $\gamma (t_1)\ll \gamma (t_2)\le \gamma (t_3)$, and hence $\gamma (t_1)\ll \gamma (t_3)$, $\gamma$ must be timelike on $[t_1,t_3]$. Then, picking some $t'<t_3$ such that $\gamma ([t',t_3])\subseteq \Omega _2$ we find ourselves in the same situation as before, only with $[t',t_3]$ replacing $[t_1,t_2]$. Consequently, we can iterate the procedure and obtain that $\gamma$ is timelike on $[t_1,b]$. Since we may symmetrically argue into the past, $\gamma$ must in fact be timelike on all of [*a*, *b*].

It remains to show that points $x\ll y$ as above always exist on $\gamma$. Since $0<\tau (p,q)=L_\tau (\gamma )$, by Lemma 2.24 it follows that $\gamma |_{[a,m]}$ or $\gamma |_{[m,b]}$ has strictly positive $\tau$-length, where $m=\frac{1}{2}(b-a)$. Iterating this bisection it follows that for any $\delta >0$ there exist $t_1<t_2$ in [*a*, *b*] such that $|t_1-t_2|<\delta$ and $\tau (\gamma (t_1),\gamma (t_2)) = L_\tau (\gamma |_{[t_1,t_2]})>0$, and so $\gamma (t_1)\ll \gamma (t_2)$. We now cover $\gamma ([a,b])$ by finitely many regularly localizing neighborhoods as in Definition 3.16 and let $\varepsilon$ be a Lebesgue number of this cover. Since $\gamma$ is uniformly continuous, by choosing $\delta$ small enough we can guarantee that $d(\gamma (t_1),\gamma (t_2))<\varepsilon$, and so both points lie in one of the neighborhoods from the cover. $\square$

**Fig. 2:**
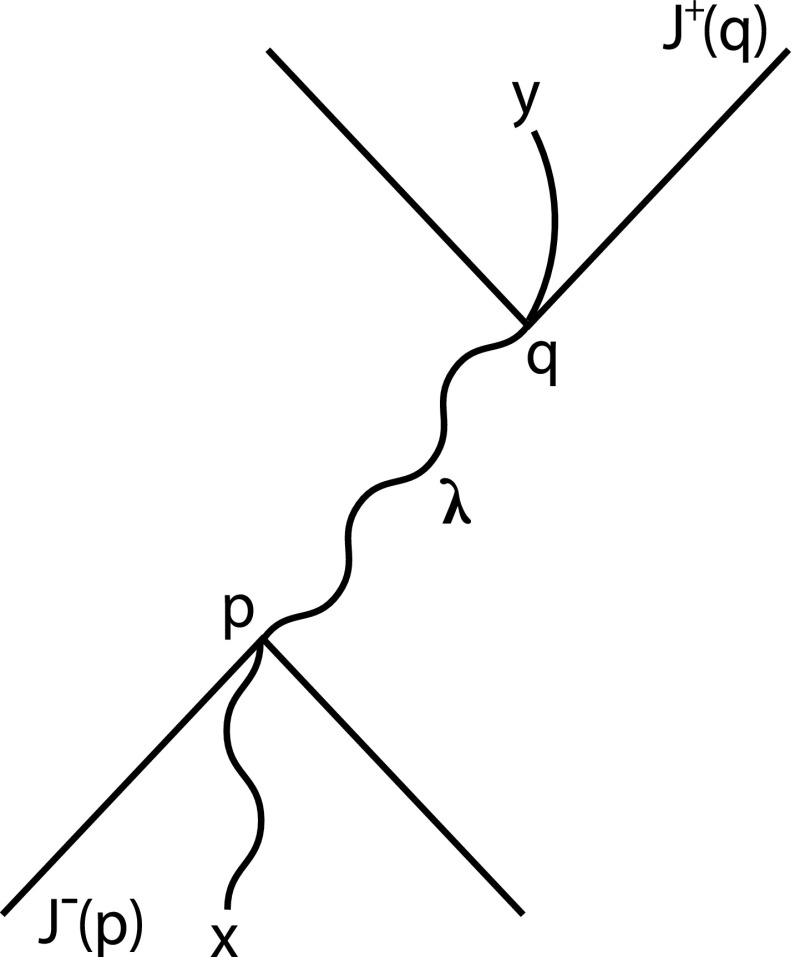
A causal funnel

#### Example 3.19

(i) *Causal funnels*
  In Minkowski space $\mathbb {R}^n_1$, let $\lambda$ be a future-directed causal curve connecting two points *p* and *q*. Let *X* be the union of $J^-(p)$, $J^+(q)$ and the image of $\lambda$ (Fig. 2).
  For $x,y\in X$, let $x\le y$ if *x* can be connected to *y* within *X* by a curve that is future-directed causal in $\mathbb {R}^n_1$, and let $x\ll y$ if this curve can be chosen to contain a timelike segment. Define $\tau (x,y)$ to be the supremum over all lengths of such curves connecting *x* and *y* if such curves exist, and 0 otherwise. Also, let *d* be the restriction of the standard metric on $\mathbb {R}^n$. Then, it is easily verified that $(X,d,\ll ,\le ,\tau )$ is a Lorentzian pre-length space. If $\lambda$ is null (hence also null in the sense of Definition 2.18), $x\ll p$, and $q\le y$, then the maximal curve from *x* to *y* necessarily changes its causal character.
(ii) As can be seen from Corollary 5.5, even in spacetimes with continuous metrics it can happen that maximal causal curves change their causal character.

We next turn to a fundamental property of smooth spacetimes, namely the push-up principle (cf. [13]): Causal curves that connect timelike-related points and contain a null segment can be deformed into timelike curves with the same endpoints and strictly greater length. This principle can be extended to the current setting as follows:

#### Theorem 3.20

Let $(X,d,\ll ,\le ,\tau )$ be a regularly localizable Lorentzian pre-length space, and let $\gamma : [a,b]\rightarrow X$ be a future-directed causal curve with $L_\tau (\gamma )>0$. If $\gamma |_{[c,d]}$ is null on some (non-trivial) subinterval [*c*, *d*] of [*a*, *b*], then there exists a strictly longer future-directed timelike curve $\sigma$ from $\gamma (a)$ to $\gamma (b)$. If *X* is even (SR)-localizable, then $\sigma$ can be chosen to lie in any given neighborhood of $\gamma ([a,b])$.

#### Proof

Without loss of generality we may suppose that $a<c$ and $d=b$, the other cases can be reduced to this one or proved analogously. Let $t_1:= \inf \{t \in [a,b] : L_\tau (\gamma |_{[t,b]}) = 0\}$, then $a<t_1$: Suppose, to the contrary, that $t_1=a$ and let $\Omega _{\gamma (a)}$ be a regularly localizing neighborhood of $\gamma (a)$. Then, since $L_\tau (\gamma |_{[s,t]})=\omega _{\gamma (a)}(\gamma (s),\gamma (t))$ depends continuously on *s* and *t* for *s*, *t* small, it would follow that $L_\tau (\gamma )=0$, contradicting our assumption.

Now let $\Omega$ be a regularly localizing neighborhood of $\gamma (t_1)$. Then, we can pick $t_0<t_1<t_2$ sufficiently close to $t_1$ to secure $\gamma (t_0), \gamma (t_2) \in \Omega$. Also, $\gamma (t_0)\ll \gamma (t_2)$ and $\gamma ([t_1,t_2])$ contains a null segment. Thus, we can connect $\gamma (t_0)$ to $\gamma (t_2)$ by a future-directed timelike curve $\sigma$ in $\Omega$ that is strictly longer than $\gamma |_{[t_0,t_2]}$.

Similarly to the proof of Theorem 3.18 we cover $\gamma ([t_0,b])$ by finitely many regularly localizing neighborhoods $\Omega _1=\Omega _2:=\Omega ,\Omega _3, \dots , \Omega _N$ as in Definition 3.16 and pick $t_3< \dots < t_{N}=b$ in $(t_2,b]$ such that $\gamma ([t_i,t_{i+1}])\subseteq \Omega _{i+1}$ for $i=0,\dots N-1$. Next, we choose a point *p* on $\sigma$ that lies in $\Omega _3$ and is timelike related to $\gamma (t_2)$ and concatenate $\sigma$ from *p* onward to a maximal curve from *p* to $\gamma (t_3)$ within $\Omega _3$. Iterating this procedure, we obtain a timelike curve from $\gamma (t_0)$ to $\gamma (b)$ that is strictly longer than $\gamma |_{[t_0,b]}$. Analogously, we can argue for $\gamma |_{[a,t_0]}$ to construct the claimed curve. Finally, if *X* is (SR)-localizable then the regularly localizing neighborhoods and thereby the timelike curve constructed above can be chosen to lie within any prescribed neighborhood of the image of $\gamma$. $\square$

Recalling Lemma 2.30, we obtain the following generalization of [13, Cor. 2.4.16]:

#### Corollary 3.21

Let $(X,d,\ll ,\le ,\tau )$ be a regularly localizable Lorentzian pre-length space, and let $\gamma : [a,b]\rightarrow X$ be a future-directed causal curve such that for some $a\le c<d\le b$, $\gamma |_{[c,d]}$ is rectifiable. Then, there exists a timelike future-directed curve from $\gamma (a)$ to $\gamma (b)$. If *X* is even (SR)-localizable, then this curve can be chosen to lie in any given neighborhood of $\gamma ([a,b])$.

### Lorentzian length spaces

Finally, we have the concepts at hand to define the following notion of *intrinsic* time separation function.

#### Definition 3.22

Let $(X,d,\ll ,\le ,\tau )$ be a locally causally closed, causally path connected and localizable Lorentzian pre-length space and let $x,y\in X$. Then set

$$\begin{aligned} {\mathcal {T}}(x,y):= \sup \Big \{L_\tau (\gamma ):\gamma \text { future-directed causal from }x \text { to } y\Big \}, \end{aligned}$$

if the set of future-directed causal curves from *x* to *y* is not empty. Otherwise set ${\mathcal {T}}(x,y):=0$. We call *X* a *Lorentzian length space* if ${\mathcal {T}} = \tau$. If, in addition, *X* is regularly localizable, then it is called a *regular* Lorentzian length space.

#### Remark 3.23

(i) The above definition is a close analogue of the notion of length spaces in metric geometry: A metric space (*X*, *d*) is a length space if for any points $x,y\in X$, *d*(*x*, *y*) equals the infimum over the length of all paths connecting them, where the length of a path is defined as the supremum of the lengths of inscribed polygons (cf. [3, 42]).
(ii) Since a Lorentzian length space is causally path connected, the set of all future-directed causal curves connecting two causally related points is never empty.
(iii) In any Lorentzian pre-length space, ${{\mathcal {T}}}(x,y)\le \tau (x,y)$ for all $x, y\in X$: This is obvious if ${\mathcal {T}}(x,y)=0$. If, on the other hand, ${\mathcal {T}}(x,y)>0$, then for any $\varepsilon >0$ there exists a future-directed causal curve $\gamma$ from *x* to *y* with ${\mathcal {T}}(x,y)< L_\tau (\gamma )+\varepsilon \le \tau (x,y)+\varepsilon$.

#### Example 3.24

(i) Let $(M,d^h,\ll ,\le ,\tau )$ be the Lorentzian pre-length space induced by a smooth and strongly causal spacetime (*M*, *g*), see Example 2.11. Then by Proposition 2.32, we know that $L_\tau = L_g$, the usual Lorentzian length functional. Thus, the definition of ${\mathcal {T}}$ is the same as for the time separation function $\tau$ of (*M*, *g*), cf. [41, Def. 14.15]. Using the exponential map and convex neighborhoods, it is not hard to see that $(M,d^h,\ll ,\le ,\tau )$ is also locally causally closed and regularly localizable. Moreover, causal path-connectedness holds due to the definition of the causal relations. Consequently, $(M,d^h,\ll ,\le ,\tau )$ is a regular and (SR)-localizable Lorentzian length space.
(ii) As in Example 3.19 (i), let *X* be a causal funnel. If the connecting curve $\lambda$ is timelike, then $(X,d,\ll ,\le ,\tau )$ is causally path connected and causally closed. It then readily follows that $(X,d,\ll ,\le ,\tau )$ is a strongly localizable Lorentzian length space.
(iii) In Sect. 5, we will give further examples of Lorentzian length spaces, and in particular we will show that spacetimes of low regularity can be viewed as Lorentzian length spaces, although not necessarily as *regular* Lorentzian length spaces. This connection is our motivation for the terminology introduced after Definition 3.16 (iv).

#### Lemma 3.25

In a Lorentzian length space , two timelike-related points can always be connected via a causal curve of positive $\tau$-length.

#### Proof

Let $(X,d,\ll ,\le ,\tau )$ be a Lorentzian length space and let $x,y\in X$ with $x\ll y$. Then, $0<\tau (x,y)={\mathcal {T}}(x,y)$. Moreover, for every $\varepsilon >0$ there is a future-directed causal curve $\gamma$ from *x* to *y* such that $L_\tau (\gamma ) > {\mathcal {T}}(x,y) - \varepsilon$. By choosing $\varepsilon =\frac{{\mathcal {T}}(x,y)}{2}>0$ it follows that $L_\tau (\gamma )>0$. $\square$

### The causal ladder for Lorentzian length spaces

#### Theorem 3.26

For Lorentzian length spaces

(i) causality implies chronology,
(ii) non-total imprisonment implies causality,
(iii) strong causality implies non-total imprisonment,
(iv) strong causality is equivalent to the nonexistence of almost closed causal curves (i.e., the converse to Lemma 2.38 (iii) holds for Lorentzian length spaces), and
(v) global hyperbolicity implies strong causality.

#### Proof

Let $(X,d,\ll ,\le ,\tau )$ be a Lorentzian length space, which for brevity we just denote by *X*. Lemma 3.2 shows that *X* is interpolative.

(i) Let *X* be causal, then Lemma 2.38 (i) establishes that *X* is chronological.
(ii) Let *X* be non-totally imprisoning. Assume that *X* is not causal, then by Lemma 3.3 (ii) we know that there is a closed causal curve $\gamma :[a,b]\rightarrow X$. Since $\gamma$ is not constant we have that $L^d(\gamma )>0$. By going infinitely often around this loop, we get a causal curve ${\tilde{\gamma }}$ such that $L^d({\tilde{\gamma }})=\infty$ and whose image is contained in the compact set $\gamma ([a,b])$—a contradiction to *X* being non-totally imprisoning.
(iii) Let *X* be strongly causal and assume that *X* is totally imprisoning. By Corollary 3.15, this means that there is a compact set $K\Subset X$ and an inextendible (future-directed) causal curve $\gamma :[0,\infty )\rightarrow X$ contained in *K*. Moreover, by Lemma 3.12 we know that $\lim _{t\nearrow \infty }\gamma (t)$ does not exist. However, for any sequence that convergences to $\infty$ there is a convergent subsequence $(t_n)_n$ with $t_n\nearrow \infty$ and $\lim _{n\rightarrow \infty }\gamma (t_n)=:p$, since $\gamma$ is contained in the compact set *K*. Now, since $\lim _{t\nearrow \infty }\gamma (t)$ does not exist there is another sequence $(s_n)_n$ with $s_n\nearrow \infty$ with $\lim _{n\rightarrow \infty }\gamma (s_n)=:q\ne p$. Let *U* be a neighborhood of *p* that does not contain *q*. By strong causality and Lemma 2.38 (iii), there exists a neighborhood *V* of *p* with $V\subseteq U$ and such that any causal curve with endpoints in *V* is contained in *U*. There is an $n_0\in \mathbb {N}$ such that $\gamma (t_n)\in V$ for all $n\ge n_0$. By mixing the sequences $(t_n)_n$ and $(s_n)_n$ to get a strictly monotonically increasing sequence $(r_n)_n$ one can find $n_1< n_2 < n_3$ such that $\gamma (r_{n_1}),\gamma (r_{n_3})\in V$ and $\gamma (r_{n_2})\notin U$. This is a contradiction since $\gamma |_{[t_{r_1},t_{r_3}]}$ is a causal curve with endpoints in *V* that leaves *U*.
(iv) Let *X* be such that for all $x\in X$, for every neighborhood *U* of *x*, there is a neighborhood $V\subseteq U$ of *x* such that for every causal curve $\gamma :[a,b]\rightarrow X$ with $\gamma (a),\gamma (b)\in V$ one has $\gamma ([a,b])\subseteq U$. Assume to the contrary that *X* is not strongly causal, i.e., there is a $p\in X$ and a $\delta >0$ such that for all $A\in {\mathcal {A}}$ (the subbase for the Alexandrov topology, cf. Sect. 2.2) with $p\in A$ one has $A\not \subseteq B^d_\delta (p)$. Now the assumptions yield that there is a *d*-neighborhood *V* of *p*, $V\subseteq B^d_\delta (p)$ such that all causal curves with endpoints in *V* are contained in $B^d_\delta (p)$. Let $\Omega$ be a localizing neighborhood for *p*, then $I^\pm (p)\cap \Omega \ne \emptyset$, thus by causal path-connectedness there is a timelike curve $\gamma$ through *p*. Now choose $p^-,p^+\in \gamma ([a,b])\cap V$ with $p^-\ll p\ll p^+$. Then, $p\in I^+(p^-)\cap I^-(p^+)\in {\mathcal {A}}$ but on the other hand $I^+(p^-)\cap I^-(p^+)\subseteq B^d_\delta (p)$—a contradiction.
(v) Let *X* be globally hyperbolic and assume that *X* is not strongly causal, i.e., there is a point $x\in X$ and a neighborhood *U* of *x* such that for all neighborhoods *V* of *x* with $V\subseteq U$ there is a causal curve with endpoints in *V* that leaves *U*. As above there is a timelike curve $\lambda$ through *x*; hence, we can choose $p,q\in U$ on $\lambda$ with $p\ll x \ll q$. Moreover, since $I^+(p)\cap I^-(q)$ is open there is a $\delta _0>0$ such that for all $0<\delta \le \delta _0$ we have $B^d_\delta (x)\subseteq I^+(p)\cap I^-(q) \subseteq J^+(p)\cap J^-(q)$, which is compact by assumption. Let $n_0\in \mathbb {N}$ with $\frac{1}{n_0}< \delta _0$, then for all $n\ge n_0$ there is a future-directed causal curve $\gamma _n:[a_n,b_n] \rightarrow X$ with $\gamma _n(a),\gamma _n(b)\in B^d_{1/n}(x)$, $\gamma _n([a_n,b_n])\subseteq J(p,q)$ and $\gamma _n([a_n,b_n])\not \subseteq U$. Thus, we can apply the limit curve theorem 3.7 to obtain a closed causal curve (which is not constant since it leaves *U*)—a contradiction to non-total imprisonment via point (ii).

$$\square $$

### Geodesic length spaces

#### Definition 3.27

A Lorentzian pre-length space $(X,d,\ll ,\le ,\tau )$ is called *geodesic* if for all $x,y\in X$ with $x<y$ there is a future-directed causal curve $\gamma$ from *x* to *y* with $\tau (x,y)=L_\tau (\gamma )$ (hence maximizing).

#### Theorem 3.28

Let $(X,d,\ll ,\le ,\tau )$ be a globally hyperbolic Lorentzian length space, then $\tau$ is finite and continuous.

#### Proof

First we show that $\tau$ is continuous. For the moment we assume that $\tau (p,q)<\infty$. Also assume to the contrary that $\tau$ is not upper semicontinuous at $(p,q)\in X\times X$. Thus, there exist some $\delta >0$ and sequences $p_n \rightarrow p$, $q_n\rightarrow q$ such that

11 $$\begin{aligned} \tau (p_n,q_n)\ge \tau (p,q) + \delta , \end{aligned}$$

for all $n\in \mathbb {N}$. Since $\tau (p,q)\ge 0$ we have that $\tau (p_n,q_n)>0$ and hence $p_n\ll q_n$ for all $n\in \mathbb {N}$. Furthermore, for $n\ge 1$ there is a future-directed causal curve $\gamma _n$ from $p_n$ to $q_n$ with $L_\tau (\gamma _n) > {\mathcal {T}}(p_n,q_n)- \frac{1}{n} = \tau (p_n,q_n)-\frac{1}{n}$. Note that, by strong causality, this shows that $p\ne q$. Again by strong causality and localizability, there are $p_-,q_+\in X$ such that $p\in I^+(p_-)$ and $q\in I^-(q_+)$. So there is an $n_0\in \mathbb {N}$ such that $p_n,q_n\in J(p_-,q_+)$ for all $n\ge n_0$. By global hyperbolicity $J(p_-,q_+)$ is compact and the image of $\gamma _n$ is contained in $J(p_-,q_+)$ for all $n\ge n_0$ and so by the Limit curve theorem 3.7 and $p\ne q$ we get that there is a subsequence $(\gamma _{n_k})_k$ of $(\gamma _n)_n$ that converges uniformly to a future-directed causal curve $\gamma$ from *p* to *q*. Moreover, by construction and the upper semicontinuity of $L_\tau$ (Proposition 3.17) this yields that

$$\begin{aligned} {\mathcal {T}}(p,q)&\ge L_\tau (\gamma )\ge \limsup _k L_\tau (\gamma _{n_k})\\&\ge \limsup _k \Big (\tau (p_{n_k},q_{n_k}) - \frac{1}{n_k}\Big ) \ge \tau (p,q) + \delta = {\mathcal {T}}(p,q) + \delta > {\mathcal {T}}(p,q), \end{aligned}$$

a contradiction. In the case that $\tau (p,q)=\infty$, there is nothing to do. Thus, $\tau$ is continuous.

It remains to show that $\tau$ is finite. Note that $(X,d,\ll ,\le ,\tau )$ is chronological by Theorem 3.26 and so $\tau (x,x)=0$ for all $x\in X$ by Lemma 2.38 (ii). Let $p,q\in X$. If $\tau (p,q)=0$, there is nothing to prove. Otherwise, there is a causal curve $\gamma :[a,b]\rightarrow X$ from *p* to *q*. The map $\Delta : [a,b] \rightarrow [0,\infty ]$, $\Delta (t)= \tau (p,\gamma (t))$ is uniformly continuous. Therefore, we may choose a partition $(t_i)_{i=0}^N$ of [*a*, *b*] such that $|\tau (p,\gamma (t_{i+1}))-\tau (p,\gamma (t_i))|<1$ for each $i\in \{0,\dots ,N\}$. Since, furthermore, $\tau$ vanishes on the diagonal, we obtain

$$\begin{aligned} \tau (p,q) = \sum _{i=0}^{N-1} \Big (\tau (p,\gamma (t_{i+1})) - \tau (p,\gamma (t_i))\Big ) \le N, \end{aligned}$$

establishing that $\tau$ is finite. $\square$

#### Remark 3.29

Finiteness of $\tau$ precludes the pathological situation where a maximal curve could have infinite length.

Finally, we obtain the following generalization of the Avez–Seifert theorem:

#### Theorem 3.30

Any globally hyperbolic Lorentzian length space $(X,d,\ll ,\le ,\tau )$ is geodesic.

#### Proof

By Theorem 3.28, we know that $\tau$ is finite and continuous. Let $x,y\in X$ with $x< y$, then $\tau (x,y)<\infty$ and we get a sequence $(\gamma _n)_n$ of future-directed causal curves $\gamma _n:[a,b]\rightarrow X$ from *x* to *y* such that $L_\tau (\gamma _n)\rightarrow \tau (x,y)$. These curves are all contained in the compact set *J*(*x*, *y*), and so by the Limit curve theorem 3.7 we get a limit curve $\gamma$ from *x* to *y* with $L_\tau (\gamma ) = \limsup _n L_\tau (\gamma _n) = \lim _n L_\tau (\gamma _n) = \tau (x,y)$. Thus, $\gamma$ is a maximal future-directed causal curve from *x* to *y*. $\square$

### Parametrization by arclength

We will now establish that a rectifiable curve (which is timelike) can be parametrized with respect to $\tau$-arclength. The only drawback is that this parametrization need not be Lipschitz continuous. Thus, the resulting curve will not be a causal curve in the sense of Definition 2.18. To handle this issue, we introduce the following notion.

#### Definition 3.31

Let $(X,d,\ll ,\le ,\tau )$ be a Lorentzian pre-length space , and let $\gamma :[a,b]\rightarrow X$ be a future-directed causal curve. A *weak parametrization* of $\gamma$ is a curve of the form $\gamma \circ \phi :[c,d]\rightarrow X$, where $\phi :[c,d]\rightarrow [a,b]$ is continuous and strictly monotonically increasing.

Note that if $\phi$ is Lipschitz continuous, then $\gamma \circ \phi$ is Lipschitz continuous, hence a causal curve. Moreover, the $\tau$-length of a weak parametrization can be defined as in Definition 2.24, and Lemma 2.28 shows that the $\tau$-length is invariant under such a reparametrization, too.

#### Lemma 3.32

Let $(X,d,\ll ,\le ,\tau )$ be a Lorentzian pre-length space, let $\gamma :[a,b]$ $\rightarrow X$ be a future-directed causal curve, and let $\lambda :=\gamma \circ \phi :[c,d] \rightarrow X$ be a weak parametrization of $\gamma$. Then, $\lambda$ has the same causal character as $\gamma$.

#### Proof

We show the case when $\gamma$ is causal, the timelike case is completely analogous. Let $c\le t_1 < t_2 \le d$, then $a\le \phi (t_1)<\phi (t_2)\le b$ and so $\lambda (t_1)=\gamma (\phi (t_1))\le \gamma (\phi (t_2)) = \lambda (t_2)$. $\square$

#### Lemma 3.33

Let $(X,d,\ll ,\le ,\tau )$ be a Lorentzian pre-length space , and let $\gamma :[a,b]\rightarrow X$ be a future-directed causal curve with $L:=L_\tau (\gamma )<\infty$. Then, the map $\phi :[a,b]\rightarrow [0,L]$, $t\mapsto L_\tau (\gamma |_{[a,t]})$ is monotonically increasing. Moreover, if the time separation function $\tau$ is continuous and satisfies $\tau (x,x)=0$ for all $x\in X$ then $\phi$ is continuous.

#### Proof

First we show that $\phi$ is monotonically increasing. Let $a\le s < t \le b$ and let $a=t_0<t_1<\cdots <t_N=t$ be a partition of [*a*, *t*]. If there is a $k\in \{1,\ldots ,N\}$ such that $t_k = s$, then $(t_i)_{i=0}^k$ is a partition of [*a*, *s*] and thus $\phi (s) \le \sum _{i=0}^{k-1}\tau (\gamma (t_i),\gamma (t_{i+1})) \le \sum _{i=0}^{N-1}\tau (\gamma (t_i),\gamma (t_{i+1}))$ On the other hand, if there is no such *k*, define $j:=\max \{1\le i \le N: t_i< s\}$. Then, $(t_i)_{i=0}^j \cup \{s\}$ is a partition of [*a*, *s*]. This yields $\phi (s) \le \sum _{i=0}^{j-1}\tau (\gamma (t_i),\gamma (t_{i+1})) + \tau (\gamma (t_j),\gamma (s)) \le \sum _{i=0}^{N-1}\tau (\gamma (t_i),\gamma (t_{i+1}))$, where we again used that $\tau \ge 0$ and the reverse triangle inequality. Taking the infimum over all partitions of [*a*, *t*] gives $\phi (s)\le L_\tau (\gamma |_{[a,t]})=\phi (t)$.

To show continuity of $\phi$ at any $t\in [a,b]$, we make use of the continuity of the maps $y\mapsto \tau (x,y)$ and $y\mapsto \tau (y,x)$ for $x\in X$ fixed. Let $t\in [a,b]$ and $\varepsilon >0$, then there is a neighborhood *U* of $\gamma (t)$ in *X* such that for all $y\in U$

12 $$\begin{aligned} \tau (\gamma (t),y)< \varepsilon \quad \text { and } \quad \tau (y,\gamma (t))<\varepsilon , \end{aligned}$$

since $\tau \ge 0$ and $\tau (\gamma (t),\gamma (t))=0$. By the continuity of $\gamma$, there is a $\delta >0$ such that $\gamma ((t-\delta ,t+\delta ))\subseteq U$. For $s\in (t-\delta ,t]$, we have by Lemma 2.25 and (12)

$$\begin{aligned} |\phi (t)-\phi (s)|= L_\tau (\gamma |_{[s,t]}) \le \tau (\gamma (s),\gamma (t))<\varepsilon . \end{aligned}$$

Analogously for $s\in [t,t+\delta )$, we have $|\phi (t)-\phi (s)| = L_\tau (\gamma |_{[t,s]}) \le \tau (\gamma (t),\gamma (s)) < \varepsilon$. $\square$

#### Proposition 3.34

Let $(X,d,\ll ,\le ,\tau )$ be a Lorentzian pre-length space with $\tau$ continuous and $\tau (x,x)=0$ for all $x\in X$. Let $\gamma :[a,b]\rightarrow X$ be a future-directed rectifiable curve with $L:=L_\tau (\gamma )<\infty$. Then, there exists a weak parametrization ${\tilde{\gamma }}$ of $\gamma$ such that ${\tilde{\gamma }}$ is parametrized with respect to $\tau$-length, i.e., ${\tilde{\gamma }}:[0,L]\rightarrow X$ with $L_\tau ({\tilde{\gamma }}|_{[0,s]}) = s$ for all $s\in [0,L]$.

#### Proof

Define $\phi :[a,b]\rightarrow [0,L]$, $t\mapsto L_\tau (\gamma |_{[a,t]})$ as in Lemma 3.33. Then, $\phi$ is strictly monotonically increasing and continuous and thus gives rise to a weak parametrization ${\tilde{\gamma }}:= \gamma \circ \phi ^{-1}:[0,L]\rightarrow [a,b]$. Note that Lemma 2.28 applies also to weak parametrizations, and hence we conclude that $L_\tau ({\tilde{\gamma }}|_{[0,s]}) = L_\tau (\gamma |_{[a,\phi ^{-1}(s)]}) = \phi (\phi ^{-1}(s)) = s$. $\square$

#### Corollary 3.35

Let $(X,d,\ll ,\le ,\tau )$ be a Lorentzian pre-length space with $\tau$ continuous and $\tau (x,x)=0$ for all $x\in X$. Then, a maximal timelike curve $\gamma$ with finite $\tau$-length has a weak parametrization $\lambda$ such that $\tau (\lambda (s_1),\lambda (s_2)) = s_2 - s_1$ for all $s_1 < s_2$ in the corresponding interval.

#### Proof

Let $\gamma$ be timelike and maximal. Then, by Proposition 2.34 (iii) $\gamma$ is rectifiable and hence by Proposition 3.34 there is a weak parametrization $\lambda =\gamma \circ \phi ^{-1}$ on $[0,L_\tau (\gamma )]$ such that $L_\tau (\lambda |_{[0,s]}) = s$. Moreover, as noted above, Lemma 2.28 applies also to weak parametrizations; hence, we have that $L_\tau (\gamma ) = L_\tau (\lambda )$ and thus for $0\le s_1 < s_2 \le L_\tau (\gamma )$ we get

$$\begin{aligned} s_2 - s_1&= L_\tau (\lambda |_{[s_1,s_2]}) = L_\tau (\gamma |_{[\phi ^{-1}(s_1),\phi ^{-1}(s_2)]})\\&= \tau (\gamma (\phi ^{-1}(s_1)),\gamma (\phi ^{-1}(s_2))) = \tau (\lambda (s_1),\lambda (s_2)). \end{aligned}$$

$$\square $$

## Curvature bounds via triangle comparison

In close analogy to the theory of CAT(*k*)- and Alexandrov spaces, in this section we introduce spaces whose curvature is bounded above or below, in terms of triangle comparison with respect to Lorentzian model spaces of constant curvature. The comparison conditions will be formulated with respect to the time separation function $\tau$.

### Timelike geodesic triangles

We begin by considering *timelike geodesic triangles* in a Lorentzian length space and compare them to timelike geodesic triangles in a model space of constant curvature.

#### Definition 4.1

A *timelike geodesic triangle* in a Lorentzian pre-length space $(X,d,\ll ,\le ,\tau )$ is a triple $(x,y,z)\in X^3$ with $x\ll y\ll z$ such that $\tau (x,z)<\infty$ and such that the sides are realized by future-directed causal curves: there are future-directed causal curves $\alpha$ from *x* to *y*, $\beta$ from *y* to *z*, and $\gamma$ from *x* to *z* such that $L_\tau (\alpha ) = \tau (x,y)$, $L_\tau (\beta ) = \tau (y,z)$ and $L_\tau (\gamma ) = \tau (x,z)$.

The reason for merely requiring the realizing curves in the previous definition to be causal instead of timelike is that in general it may happen that maximizing curves change their causal character. For a concrete example see Corollary 5.5. By Theorem 3.18, however, such a situation cannot occur in regularly localizable pre-length spaces.

#### Lemma 4.2

Let $(X,d,\ll ,\le ,\tau )$ be a Lorentzian pre-length space and let (*x*, *y*, *z*) be a geodesic triangle. Let $\alpha , \beta , \gamma$ be future-directed causal curves from *x* to *y*, *y* to *z*, and *x* to *z*, respectively, such that $L_\tau (\alpha ) = \tau (x,y)=:a$, $L_\tau (\beta ) = \tau (y,z)=:b$ and $L_\tau (\gamma ) = \tau (x,z)=:c$. Then, $a<\infty$, $b<\infty$ and $\alpha , \beta , \gamma$ are maximal.

#### Proof

By the reverse triangle inequality, we get $a = \tau (x,y)< \tau (x,y) + \tau (y,z) \le \tau (x,z)<\infty$. Analogously one shows that $b<\infty$. Let $\alpha$ be defined on the interval $[t_0,t_1]$, then $L_\tau (\alpha ) = \tau (x,y) =\tau (\alpha (t_0),\alpha (t_1))$, hence $\alpha$ is maximal. Similarly, $\beta$ and $\gamma$ are maximal. $\square$

#### Remark 4.3

In the situation of the previous Lemma, if $\tau$ is continuous and $\tau (x,x)=0$ for all *x*, then for any $0< s < \tau (x,y)$ there is a point *q* on the image of $\alpha$ with $\tau (x,q)=s$ (and analogously for $\beta$ and $\gamma$). In fact, let $\alpha : [a,b]\rightarrow X$. Then since $t\mapsto \tau (x,\alpha (t))$ is continuous, it attains any value between $0=\tau (x,\alpha (a))$ and $\tau (x,y)=\tau (x,\alpha (b))$.

Moreover, if $\alpha$, $\beta$, $\gamma$ are timelike then Corollary 3.35 allows one to obtain weak parametrizations of $\alpha , \beta , \gamma$ with respect to $\tau$-length.

#### Lemma 4.4

Let $(X,d,\ll ,\le ,\tau )$ be a globally hyperbolic Lorentzian length space, then any triple of points $(x,y,z)\in X^3$ with $x\ll y\ll z$ is a geodesic timelike triangle, whose sides, if timelike, can be weakly parametrized with respect to $\tau$-length.

#### Proof

By Theorem 3.28 $\tau$ is finite and continuous, implying in particular $\tau (x,z)<\infty$. By Theorem 3.26, we know that *X* is chronological, and thus $\tau$ is zero on the diagonal by Lemma 2.38 (ii). Furthermore, by Theorem 3.30 *X* is geodesic; hence, there are maximal causal curves realizing the sides of the triangle, which, if timelike, can be weakly parametrized with respect to $\tau$-length by Corollary 3.35. $\square$

### Model spaces of constant curvature

Curvature bounds for Lorentzian length spaces will be based on triangle comparison in relation to model spaces of constant curvature. In the present section, we introduce these model spaces, following [1, 24].

#### Definition 4.5

Let $K\in \mathbb {R}$. By $M_K$, we denote the simply connected two-dimensional Lorentz space form of constant curvature *K*.

Following the notation of [41, Ch. 8], we have

$$\begin{aligned} M_K = \left\{ \begin{array}{ll} {{\tilde{S}}}^2_1(r) &{}\quad K=\frac{1}{r^2}\\ \mathbb {R}^2_1 &{}\quad K=0\\ {{\tilde{H}}}^2_1(r) &{}\quad K= -\frac{1}{r^2}. \end{array} \right. \end{aligned}$$

Here, ${{\tilde{S}}}^2_1(r)$ is the simply connected covering manifold of the two-dimensional Lorentzian pseudosphere $S^2_1(r)$, $\mathbb {R}^2_1$ is two-dimensional Minkowski space, and ${{\tilde{H}}}^2_1(r)$ is the simply connected covering manifold of the two-dimensional Lorentzian pseudohyperbolic space.

Concerning the existence of comparison triangles in the model spaces, we may directly utilize the *Realizability Lemma* [1, Lemma 2.1] to obtain conditions on a triple (*a*, *b*, *c*) to be realized as the side lengths of a timelike triangle in a model space $M_K$. Below, we will set $\frac{\pi }{\sqrt{K}}:=\infty$ if $K\le 0$.

#### Lemma 4.6

(Realizability) Let $K\in \mathbb {R}$. Let $(a,b,c)\in \mathbb {R}_+^3$ with $c\ge a+b$. If $c=a+b$, then let $c<\frac{\pi }{\sqrt{K}}$. If $c>a+b$ and $K<0$, then assume $c<\frac{\pi }{\sqrt{-K}}$. Then, there exists a timelike geodesic triangle in $M_K$ with side lengths *a*, *b*, *c*.

#### Proof

To deduce this from [1, Lemma 2.1], note that in [1] lengths are always signed. Since we consider only timelike geodesic triangles (and unsigned lengths), (*a*, *b*, *c*) corresponds to $(-a,-b,-c)$ in [1]. The result can then immediately be read off from [1, Lemma 2.1, points 2. and 3.]. $\square$

Following [1], a triple (*a*, *b*, *c*) as in the assumptions of Lemma 4.6 will be said to *satisfy timelike size bounds* for *K*.

### Timelike curvature bounds

To concisely formulate our notion of timelike curvature bounds in Lorentzian pre-length spaces, we introduce the following terminology: Let (*x*, *y*, *z*) be a timelike geodesic triangle in a Lorentzian pre-length space as in Definition 4.1, realized by maximal causal curves $\alpha , \beta , \gamma$, and suppose that $({\bar{x}},{\bar{y}},{\bar{z}})$ is a timelike geodesic triangle in a model space $M_K$ with identical side lengths realized by (necessarily) timelike geodesics ${\bar{\alpha }}$, $\bar{\beta }$, $\bar{\gamma }$. Denote the time separation function in $M_K$ by $\bar{\tau }$. We say that a point *q* on $\alpha$ *corresponds* to a point ${{\bar{q}}}$ on $\bar{\alpha }$ if $\tau (x,q)=\bar{\tau }({{\bar{x}}}, {{\bar{q}}})$, and analogously for $\beta$ and $\gamma$. By Remark 4.3, under the assumptions made in the following Definition, any intermediate value of $\tau$ along $\alpha , \beta , \gamma$ is actually attained.

#### Definition 4.7

**Fig. 3:**
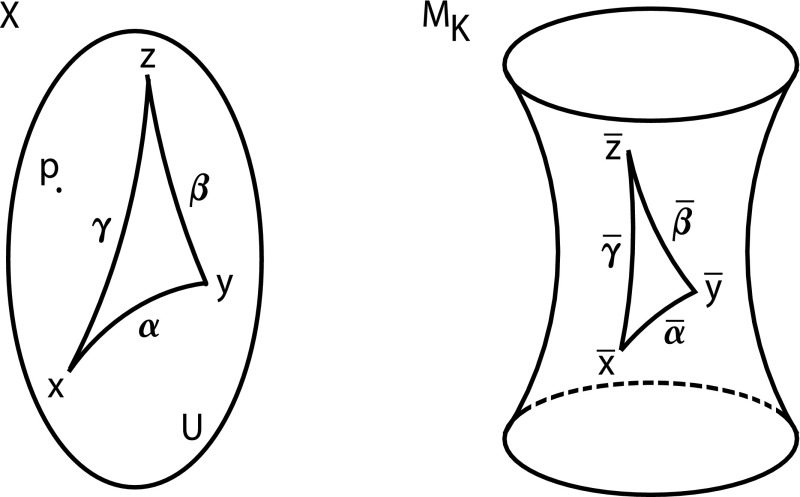
Timelike triangle in *X* and comparison triangle in $M_K$

A Lorentzian pre-length space $(X,d,\ll ,\le ,\tau )$ has timelike curvature bounded below (above) by $K\in \mathbb {R}$ if every point in *X* possesses a neighborhood *U* such that:

(i) $\tau |_{U\times U}$ is finite and continuous.
(ii) Whenever *x*, $y \in U$ with $x\ll y$, there exists a future-directed causal curve $\alpha$ in *U* with $L_\tau (\alpha ) = \tau (x,y)$.
(iii) Let (*x*, *y*, *z*) be a timelike geodesic triangle in *U*, realized by maximal causal curves $\alpha , \beta , \gamma$ whose side lengths satisfy timelike size bounds for *K*, and let $({\bar{x}},{\bar{y}},{\bar{z}})$ be a comparison triangle of (*x*, *y*, *z*) in $M_K$ as given by Lemma 4.6, realized by timelike geodesics ${\bar{\alpha }}$, $\bar{\beta }$, $\bar{\gamma }$. Then whenever *p*, *q* are points on the sides of (*x*, *y*, *z*) and ${{\bar{p}}}$, ${{\bar{q}}}$ are corresponding points of the sides of $({\bar{x}},{\bar{y}},{\bar{z}})$, we have $\tau (p,q)\le \bar{\tau }({{\bar{p}}}, {{\bar{q}}})$ (respectively, $\tau (p,q)\ge \bar{\tau }({{\bar{p}}}, {{\bar{q}}}))$ (Fig. 3).

Such a neighborhood *U* is called *comparison neighborhood with respect to* $M_K$.

#### Remark 4.8

(i) The above definition is as close as possible to the corresponding definition of curvature bounds in its metric analogue, the theory of CAT(*k*), respectively, Alexandrov spaces [3, Def. 4.1.9, Def. 9.1.1].
(ii) Condition 1 of Definition 4.7 in particular secures that $\tau (x,x)=0$ for every $x\in X$ via Proposition 2.14, so Remark 4.3 applies.

#### Example 4.9

In [1], sectional curvature bounds for general semi-Riemannian manifolds were introduced. A smooth Lorentzian manifold *M* is defined to satisfy a lower sectional curvature bound $R\ge K$ if spacelike sectional curvatures are $\ge K$ and timelike sectional curvatures are $\le K$ (and $R\le K$ with “timelike” and “spacelike” reversed). It then follows from [1, Prop. 5.2] that $R\ge K$ (respectively, $R\le K$) in this sense implies that *M* has timelike curvature bounded below (respectively, above) by *K* in the sense of Definition 4.7. Hence, a smooth strongly causal Lorentzian manifold with $R\ge K$ in the sense of [1], while having timelike sectional curvature bounded *above* by *K* has timelike sectional curvature bounded *below* by *K* in the sense of Definition 4.7, and analogously for $R\le K$.

### Branching of maximal curves

#### Definition 4.10

(*Definition of a branching point*) Let $(X,d,\ll ,\le ,\tau )$ be a Lorentzian pre-length space , and let $\gamma :[a,b]\rightarrow X$ be a maximal causal curve. A point $x:=\gamma (t)$ with $t\in (a,b)$ is called *branching point* of $\gamma$ if there exist maximal causal curves $\alpha ,\beta :[a,c]\rightarrow X$ with $c>b$ such that $\alpha |_{[a,t]} = \beta |_{[a,t]} = \gamma |_{[a,t]}$ and $\alpha ([t,c])\cap \beta ([t,c]) = \{x\}$. If $\alpha , \beta , \gamma$ are timelike then *x* is called a timelike branching point.

#### Example 4.11

(i) In a causal funnel (see Examples 3.19 and 3.24), any maximal causal curve from $J^-(p)$ to $J^+(q)$ has *q* as a branching point.
(ii) For an example of branching in the setting of spacetimes with continuous Lorentzian metrics, see Corollary 5.5.

In preparation for the following result, call a Lorentzian pre-length space timelike locally uniquely geodesic (l.u.g.) if every point $x\in X$ has a neighborhood such that, if $p\ll q$ and $p,q\in U$ then there exists a unique maximal future-directed causal curve from *p* to *q* in *U*. Hence, compared to (ii) of 4.7 one additionally requires uniqueness. Already for low regularity Lorentzian metrics, timelike l.u.g. and non-branching are independent properties. In fact, the classical paper [26] contains examples of ${\mathcal {C}}^1$-Riemannian metrics that are locally uniquely geodesic but display branching, as well as non-branching metrics that fail to be locally uniquely geodesic. These examples can be translated into the Lorentzian setting, cf. [51].

Contrary to the case of metric spaces, in the Lorentzian setting the fact that the time separation function satisfies the reverse triangle inequality precludes a direct way of generating non-degenerate triangles (i.e., such that the strict triangle inequality holds for $\tau$ on their vertices), as required in the standard proof of non-branching under lower curvature bounds (cf. [50, Lemma 2.4]). Conditions (i) and (ii) of the following theorem are sufficient to exclude degeneracy of comparison triangles.

#### Theorem 4.12

Let $(X,d,\ll ,\le ,\tau )$ be a strongly causal Lorentzian pre-length space with timelike curvature bounded below by some $K\in \mathbb {R}$ such that either

(i) Any point in *X* has a relatively compact, causally closed neighborhood $\Omega$ such that for any $p\ll q$ in $\Omega$ there is a maximal future-directed timelike curve from *p* to *q* in $\Omega$ that is strictly longer than any future-directed causal curve from *p* to *q* in $\Omega$ that contains a null segment, and there is some $C>0$ bounding the *d*-length of any causal curve in $\Omega$ (cf. (i) and (iv) from Definition 3.16), or
(ii) *X* is timelike locally uniquely geodesic.

Then, maximal timelike curves in *X* do not have timelike branching points.

#### Proof

Assume there is a (without loss of generality future-directed) maximal timelike curve $\lambda :[a,b]\rightarrow X$ that has a timelike branching point $x=\lambda (t_0)$ ($t_0\in (a,b)$). Then, there are future-directed timelike maximal curves $\alpha ,\beta :[a,c]\rightarrow X$ with $c>b$ such that $\alpha (t_0) = x = \beta (t_0)$, $\alpha |_{[a,t_0]} = \beta |_{[a,t_0]} = \lambda |_{[a,t_0]}$ and $\alpha ([t_0,c])\cap \beta ([t_0,c]) = \{x\}$. Let *U* be an open comparison neighborhood of *x* with respect to $M_K$, and let $\Omega$ be a neighborhood of *x* as provided by either (i) or (ii). Let $V\subseteq U\cap \Omega$ be an open neighborhood of *x* such that all causal curves with endpoints in *V* are contained in $U\cap \Omega$ (cf. Lemma 2.38 (iii)).

Our first aim is to show that under any of the assumptions (i) or (ii) we can construct a non-degenerate timelike triangle (*p*, *q*, *r*) in $U\cap \Omega$, i.e., with $\tau (p,q)+\tau (q,r) < \tau (p,r)$.

Let $t'\in [a,t_0)$ such that $p:=\lambda (t')\in V$. Choose $s\in (t_0,c]$ such that $\beta ([t_0,s])\subseteq V$ and such that there exists some ${{\tilde{s}}}\in (t_0,c]$ with $\tau (p,\beta (s))=\tau (p,\alpha ({{\tilde{s}}}))$, and set $r:=\beta (s)$ and $r':=\alpha ({{\tilde{s}}})$.

Assuming (ii), note that $I^-(r)\cap V$ is an open neighborhood of *x*, thus $\alpha ^{-1}(I^-(r)\cap V)$ is an open neighborhood of $t_0$ in [*a*, *c*], so there is an $s'\in [a,c]$, $s'>t_0$ with $q:=\alpha (s')\in I^-(r)\cap V$. By our choice of *V*, we have that $\alpha ([t_0,s'])\subseteq U\cap \Omega$. Consequently, we obtain $p\ll q\ll \beta (s)=r$ and there is a unique future-directed maximal causal curve $\gamma$ of positive length from *q* to *r* in $U\cap \Omega$ by Definition 4.7,(ii). This gives a timelike geodesic triangle (*p*, *q*, *r*) contained in $U\cap \Omega$. Moreover, by the timelike l.u.g.-property of $\Omega$, the sidelengths of (*p*, *q*, *r*) satisfy the strict triangle inequality.

**Fig. 4:**
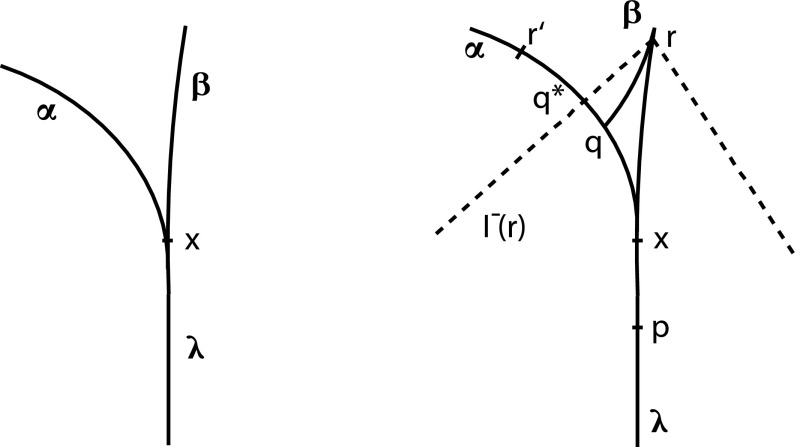
Timelike branching

Alternatively assuming (i), we first show $\tau (r',r)=0$. If $\tau (r',r)>0$ we get $\tau (p,r')<\tau (p,r') + \tau (r',r) \le \tau (p,r) = \tau (p,r')$—a contradiction. Defining $s^*:=\sup \{t\in [t',c]: \tau (\alpha (t),r)>0\}$, we obtain $s^*\le {{\tilde{s}}}$ and thus $q^*:=\alpha (s^*)\le r'$ and $\tau (q^*,r)=0$. We distinguish two cases: First, let $q^*\ll r'$. Then, $\tau (p,q^*) < \tau (p,r') = \tau (p,r)$ and we pick $\varepsilon >0$ such that $\tau (p,q^*) + \varepsilon < \tau (p,r)$. By continuity, we can choose $q\ll q^*$ on $\alpha$ with $\tau (q,r)<\varepsilon$, and hence $\tau (p,q) + \tau (q,r)< \tau (p,q^*) + \varepsilon < \tau (p,r)$ (Fig. 4). This gives the non-degenerate timelike triangle. In the second case, where $q^*=r'$ we derive a contradiction as follows. Picking a sequence $t_n\nearrow s^*$, by (i) there exists a sequence $\sigma _n$ of future-directed timelike curves in $U\cap \Omega$ from $\alpha (t_n)$ to *r*. By Theorem 3.7, $\sigma _n$ possesses a limit curve $\sigma$ that is future-directed causal and connects $r'$ to *r*. As $\tau (r',r)=0$, $\sigma$ has to be null. Then, the concatenation of $\alpha |_{[t',s*]}$ and $\gamma$ is a future-directed causal curve from *p* to *r* that contains a null segment and so by (i), there exists a strictly longer timelike curve $\chi$ from *p* to *r*. Hence,

$$\begin{aligned} \tau (p,r') + \tau (r',r) = L_\tau (\alpha |_{[t',s*]}) + L_\tau (\sigma ) < L_\tau (\chi ) \le \tau (p,r) = \tau (p,r'), \end{aligned}$$

which is impossible.

Thus, under any of the assumptions (i) or (ii) of the theorem we arrive at a non-degenerate timelike triangle, and it is clear that the points in the above constructions can be chosen in such a way that the side lengths of this triangle satisfy timelike curvature bounds.

Let ${\bar{\Delta }}:=({\bar{p}},{\bar{q}},{\bar{r}})$ be a comparison triangle in $M_K$. Denote the sides of ${\bar{\Delta }}$ by $\bar{\alpha },\bar{\beta },\bar{\gamma }$ and let ${\bar{x}}_1$ be a point on $\bar{\alpha }$ and ${\bar{x}}_2 \ne {\bar{r}}$ a point on $\bar{\beta }$ such that $\bar{\tau }({\bar{p}},{\bar{x}}_1) = \bar{\tau }({\bar{p}},{\bar{x}}_2) = \tau (p,x)>0$. Note that since ${\bar{\Delta }}$ is a non-degenerate triangle we have that ${\bar{x}}_1\ne {\bar{x}}_2$. Moreover, we have $\bar{\tau }({\bar{x}}_1,{\bar{r}})<\bar{\tau }({\bar{x}}_2,{\bar{r}})$ since otherwise the broken future-directed timelike geodesic going from ${\bar{p}}$ to ${\bar{x}}_1$ to ${\bar{r}}$ would be at least as long as the unbroken future-directed timelike geodesic $\bar{\beta }$ from ${\bar{p}}$ to ${\bar{r}}$. Finally, since the timelike curvature is bounded from below by *K* we obtain that

$$\begin{aligned} \tau (x,r) = \bar{\tau }({\bar{x}}_2,{\bar{r}}) > \bar{\tau }({\bar{x}}_1,{\bar{r}}) \ge \tau (x,r), \end{aligned}$$

a contradiction. $$\square $$

As an immediate consequence, we obtain:

#### Corollary 4.13

Let $(X,d,\ll ,\le ,\tau )$ be a strongly causal Lorentzian length space with timelike curvature bounded below by some $K\in \mathbb {R}$ that is either regular and locally compact or timelike locally uniquely geodesic. Then, maximal timelike curves in *X* do not have timelike branching points.

### Causal curvature bounds

As already indicated in Remark 4.9, Alexander and Bishop [1] introduced Alexandrov curvature bounds for smooth semi-Riemannian manifolds. In fact, they achieve a complete characterization of sectional curvature bounds in terms of triangle comparison [1, Thm. 1.1]. In their approach, the sides of any given geodesic triangle (in a sufficiently small normal neighborhood) are parametrized on the interval [0, 1], and this affine parameter is used for comparing with corresponding triangles in the model spaces. Moreover, timelike geodesics are assigned negative lengths, and spacelike geodesics positive lengths.

When trying to generalize this approach to Lorentzian pre-length spaces, there are two basic problems. First, there is no built-in notion of spacelike curve in our setting. In addition, while in the timelike case a viable substitute for a geodesic in Lorentzian manifolds is given by the notion of maximal causal curve between timelike-related points (as implemented in Definition 4.7), even for maximal null curves there is no natural parametrization. Indeed, the affine parametrizations on [0, 1] employed in the comparison results of [1] rely on the fact that geodesics satisfy a second order ODE, which already for Lorentzian metrics on spacetimes of regularity below $C^1$ has no classical counterpart, hence is also unavailable in our setting.

Nevertheless, a restricted notion of causal curvature bounds can also be implemented for Lorentzian pre-length spaces. In fact, in addition to timelike geodesic triangles in the sense of Definition 4.1 we may consider triangles (*x*, *y*, *z*) that satisfy $x\ll y \le z$ or $x \le y \ll z$ such that $\tau (x,z)<\infty$ and such that the sides (if non-trivial) are realized by future-directed causal curves. Such triangles will be called *admissible causal geodesic triangles*. Those sides of any such triangle whose vertices are timelike related are called the timelike sides of the triangle. Since one of the sides of the triangle may have vanishing $\tau$-length, when realizing such sides by maximal curves in *X*, or the comparison triangle by causal geodesics in the model spaces, we now allow for the degenerate cases where the realizing curve (either in *X* or in the model space) is in fact constant. By [1, Lemma 2.1], the realizability Lemma 4.6 only needs minor modifications to also cover the current setup: Let $(a,b,c)\in \mathbb {R}_{\ge 0}$, with $c\ge a+b$ and at most one entry equal to 0. Then, the same bounds as is Lemma 4.6 guarantee the existence of causal comparison triangles in the model spaces. With these conventions, the analogue of Definition 4.7 reads:

#### Definition 4.14

A Lorentzian pre-length space $(X,d,\ll ,\le ,\tau )$ has causal curvature bounded below (above) by $K\in \mathbb {R}$ if every point in *X* possesses a neighborhood *U* such that:

(i) $\tau |_{U\times U}$ is finite and continuous.
(ii) Whenever $x,y \in U$ with $x < y$, there exists a causal curve $\alpha$ in *U* with $L_\tau (\alpha ) = \tau (x,y)$.
(iii) Let (*x*, *y*, *z*) be an admissible causal geodesic triangle in *U*, realized by maximal causal curves (or a constant curve, respectively) $\alpha , \beta , \gamma$ whose side lengths satisfy timelike size bounds for *K*, and let $({\bar{x}},{\bar{y}},{\bar{z}})$ be a comparison triangle of (*x*, *y*, *z*) in $M_K$ realized by causal geodesics (or a constant curve, respectively) ${\bar{\alpha }}$, $\bar{\beta }$, $\bar{\gamma }$. Then whenever *p*, *q* are points on the timelike sides of (*x*, *y*, *z*) and ${{\bar{p}}}$, ${{\bar{q}}}$ are corresponding points of the timelike sides of $({\bar{x}},{\bar{y}},{\bar{z}})$, we have $\tau (p,q)\le \bar{\tau }({{\bar{p}}}, {{\bar{q}}})$ (respectively $\tau (p,q)\ge \bar{\tau }({{\bar{p}}}, {{\bar{q}}}))$.

Again such a neighborhood *U* is called a *comparison neighborhood with respect to* $M_K$.

Since as explained above there is no natural parametrization for the null side of an admissible causal geodesic triangle, there is also no natural notion of corresponding points for these sides and the null side of the comparison triangle in the model space. Thus the restriction to the timelike sides in the above definition.

Despite this limitation, causal curvature bounds make it possible to establish properties of maximal curves and of length-increasing push-up that are closely analogous to those of regularly localizable Lorentzian length spaces (cf. Theorems 3.18 and 3.20). To show these, we start with the following observation:

#### Proposition 4.15

Let $(X,d,\ll ,\le ,\tau )$ be a strongly causal Lorentzian pre-length space with causal curvature bounded above. Let $\gamma : [a,b]\rightarrow X$ be a future-directed causal curve with $\gamma (a)\ll \gamma (b)$ and suppose there exists some (non-trivial) subinterval [*c*, *d*] of [*a*, *b*] such that $\gamma |_{[c,d]}$ is null. Then, $\gamma$ is not maximal.

#### Proof

**Fig. 5:**
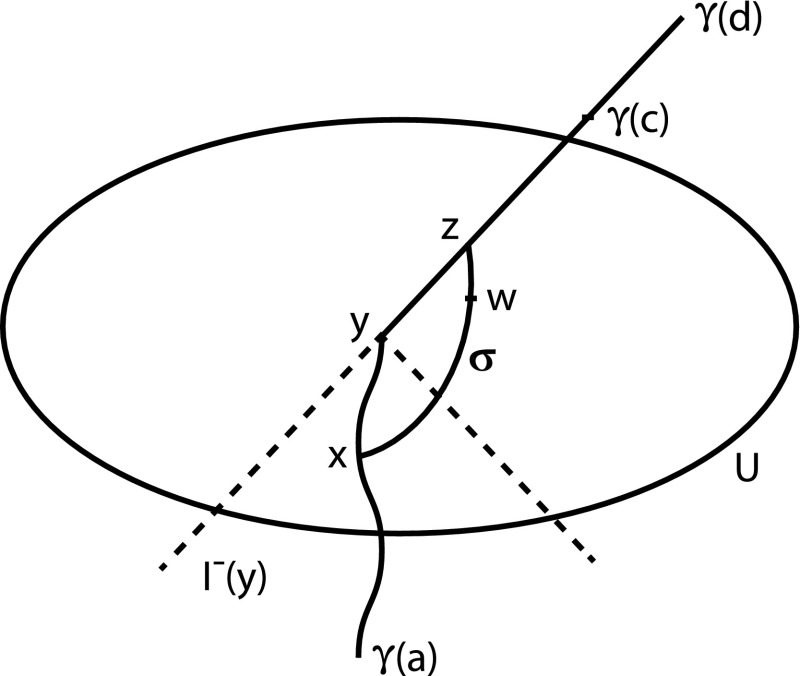
Push-up via upper causal curvature bound

It suffices to consider the case where $a<c$ and $d=b$. So suppose, to the contrary, that $\gamma$ were maximal and let $t_0:= \inf \{t \in [a,b] : L_\tau (\gamma |_{[t,b]}) = 0\}$. By Proposition 2.34 (ii) and the lower semicontinuity of $\tau$, $L_\tau (\gamma |_{[t_0,b]})=\tau (\gamma (t_0),\gamma (b))=0$. Also, $a<t_0 (\le c)$ since otherwise $\tau (\gamma (a),\gamma (b))$ would vanish, contrary to our assumption. Moreover, for any $a\le s <t_0$, $\tau (\gamma (s),\gamma (t_0))= L_\tau (\gamma |_{[s,t_0]})>0$ by definition of $t_0$, and $\gamma (s)\ll \gamma (b)$ (Fig. 5).

Pick some $x:=\gamma (s)$ and $z:=\gamma (t)$ for $s<t_0<t$ and *s*, *t* sufficiently close to $t_0$ so that both points are contained in a comparison neighborhood *U* around $y:=\gamma (t_0)$. Again by Proposition 2.34 (i), $\tau (x,z) = L_\tau (\gamma _{[s,t]})=L_\tau (\gamma _{[s,t_0]})=\tau (x,y)>0$, and $\tau (y,z) = L_\tau (\gamma _{[t_0,t]})=0$. Also, we may choose *s* and *t* such that the side lengths satisfy timelike size bounds for the upper bound *K* on the curvature. Then, the corresponding comparison triangle $({{\bar{x}}}, {{\bar{y}}}, {{\bar{z}}})$ in the model space $M_K$ is degenerate, with the timelike sides coinciding and the null side collapsing to a single point ${{\bar{y}}} = {\bar{z}}$.

Since *X* is strongly causal, we may choose a point $w < z$ on a realizing causal curve $\sigma$ from *x* to *z* that is sufficiently close to *z* so that $w\not \in I^-(y)$. However, by our assumption on the upper curvature bound we have $0< {\bar{\tau }}({\bar{w}},{\bar{y}}) \le \tau (w,y)$, a contradiction. $\square$

#### Remark 4.16

Combining Proposition 4.15 with the general assumptions on comparison neighborhoods, we obtain that in a strongly causal Lorentzian pre-length space with causal curvature bounded above, every point possesses a neighborhood in which property (iv) from Definition 3.16 is satisfied. This allows us to adapt the proofs of Theorems 3.18 and 3.20 almost verbatim to obtain the following two results.

#### Theorem 4.17

In any strongly causal Lorentzian pre-length space with causal curvature bounded above, maximal causal curves have a causal character, i.e., if for a (future-directed) maximal causal curve $\gamma :[a,b]\rightarrow X$ there are $a\le t_1 < t_2\le b$ with $\gamma (t_1)\ll \gamma (t_2)$, then $\gamma$ is timelike. Otherwise it is null.

Furthermore, also in the current setting the principle of length-increasing push-up of causal curves is valid:

#### Theorem 4.18

Let $(X,d,\ll ,\le ,\tau )$ be a strongly causal Lorentzian pre-length space with causal curvature bounded above and let $\gamma : [a,b]\rightarrow X$ be a future-directed causal curve.

(i) If $L_\tau (\gamma )>0$ and if $\gamma |_{[c,d]}$ is null on some (non-trivial) subinterval [*c*, *d*] of [*a*, *b*], then there exists a strictly longer future-directed timelike curve $\sigma$ from $\gamma (a)$ to $\gamma (b)$.
(ii) If there exist $a\le c<d\le b$ such that $\gamma |_{[c,d]}$ is rectifiable, then there exists timelike future-directed curve from $\gamma (a)$ to $\gamma (b)$.

This result provides an interesting perspective on length-increasing push-up, namely that it is a necessary consequence of any upper bound on synthetic causal curvature, while in the smooth setting it is usually traced back to the Gauss Lemma. As already pointed out in Remark 4.16, there is also a close connection to regular localizability in the sense of Definition 3.16, cf. also Example 5.13 (ii).

Another consequence of the observation in Remark 4.16 is the following corollary of Theorem 4.12.

#### Corollary 4.19

Let $(X,d,\ll ,\le ,\tau )$ be a strongly causal Lorentzian pre-length space with timelike curvature bounded below and causal curvature bounded above. Then, maximal timelike curves in *X* do not have timelike branching points.

### Curvature singularities

The synthetic approach to curvature bounds developed in the previous sections in particular allows one to define curvature singularities in Lorentzian pre-length spaces, and thereby in particular in spacetimes of low regularity, where a classical description in terms of the Riemann curvature tensor is not viable, or even in settings where there is no spacetime metric available at all. In the present section, we introduce the necessary notions.

#### Definition 4.20

A Lorentzian pre-length space $(X,d,\ll ,\le ,\tau )$ has timelike (respectively, causal) curvature unbounded below/above if every point in *X* possesses a neighborhood *U* such that 1. and 2. from Definition 4.7 (respectively, Definition 4.14) are satisfied, but such that the corresponding part of property 3 from these definitions fails to hold for any $K\in \mathbb {R}$. In this case, we say that *X* has a curvature singularity.

Thus, we assume that locally there always exist timelike triangles, but that the comparison conditions fail somewhere in *X*.

#### Example 4.21

Consider a causal funnel *X* as in Example 3.19 with $\lambda$ timelike. Since *X* is clearly timelike uniquely geodesic, Example 4.11 (i) and Theorem 4.12 imply that *X* has timelike curvature unbounded below. Moreover, if $\lambda$ is null then one easily constructs maximal curves violating Theorem 4.18 (i), so in this case the causal curvature of *X* is unbounded above.

Curvature singularities are of central importance in General Relativity. As an application of the notions introduced above, we therefore demonstrate that the central singularity of the interior Schwarzschild metric can be detected by timelike triangle comparison.

#### Example 4.22

The curvature singularity of the interior of a Schwarzschild black hole.

Consider the interior Schwarzschild metric (cf., e.g., [15, 41, 57])

13 $$\begin{aligned} ds^2 = -\left( 1-\frac{2M}{r} \right) dt^2 +\left( 1-\frac{2M}{r} \right) ^{-1} dr^2 + r^2\left( d\theta ^2 + \sin ^2\theta d\phi ^2\right) , \end{aligned}$$

where $M>0$, $t\in \mathbb {R}$, $r\in (0,2M)$ and $\theta$, $\phi$ parametrize the two-sphere $S^2$. The metric (13) has the form of a warped product, with fiber $S^2$ and leafs isometric to $\mathbb {R}\times (0,2M)$ with metric

14 $$\begin{aligned} g = -\left( \frac{2M}{r} - 1 \right) ^{-1} dr^2 +\left( \frac{2M}{r} -1 \right) dt^2. \end{aligned}$$

We write *g* in this form to emphasize that, in the Schwarzschild interior, the coordinate *r* is timelike, and *t* is spacelike. As is well known, for $r\rightarrow 0+$, this metric incurs a curvature singularity. In fact, its sectional curvature (i.e., its Gauss curvature), is given by $K=\frac{2M}{r^3}$ [41, Lemma 13.3], hence goes to $+\,\infty$ as one approaches the spacelike hypersurface $r=0$.

Although [1, Thm. 1.1] provides a characterization of sectional curvature in terms of triangle comparison, we cannot directly from that result conclude that the spacetime (14) displays a timelike curvature singularity in the sense of Definition 4.20. In fact, divergence of timelike sectional curvature (as is the case here) only implies that triangle comparison for triangles of arbitrary causal character will fail, and does not necessarily entail that *timelike* triangles will be the culprits for this behavior. Instead, to verify the conditions of Definition 4.20 we will explicitly study a family of timelike geodesic triangles approaching $r=0$.

For brevity, put $h(r):=1-\frac{2M}{r}$. Then denoting by $\tau$ proper time along a timelike geodesic, by [41, Prop. 13.11] in the case $L=0$, for the constant of motion *E* we have

15 $$\begin{aligned}&E = h(r)\frac{\mathrm{d}t}{\mathrm{d}\tau } \end{aligned}$$

16 $$\begin{aligned}&E^2 = \left( \frac{\mathrm{d}r}{\mathrm{d}\tau }\right) ^2 + h(r). \end{aligned}$$

By [15, Eq. (81)], for $E=1$ there are two families of pregeodesics $\gamma _\pm (r)=(r,t_\pm (r)+ \mathrm {const})$, where

17 $$\begin{aligned} t_\pm (r) = \pm \frac{2}{3}(6M+r)\sqrt{\frac{r}{2M}} \mp 4M \mathrm {artanh}\left( \sqrt{\frac{r}{2M}}\right) . \end{aligned}$$

Here, the corresponding proper time is given by (see [15, Eq. (76)])

18 $$\begin{aligned} \tau (r) = \pm \sqrt{\frac{2r}{M}} \frac{r}{3} + \mathrm {const}. \end{aligned}$$

Also, for $E=0$ there is a pregeodesic of the form $\gamma _0(r)=(r,0)$, with

19 $$\begin{aligned} \frac{\mathrm{d}r}{\mathrm{d}\tau }=\sqrt{\frac{2M}{r}-1}. \end{aligned}$$

**Fig. 6:**
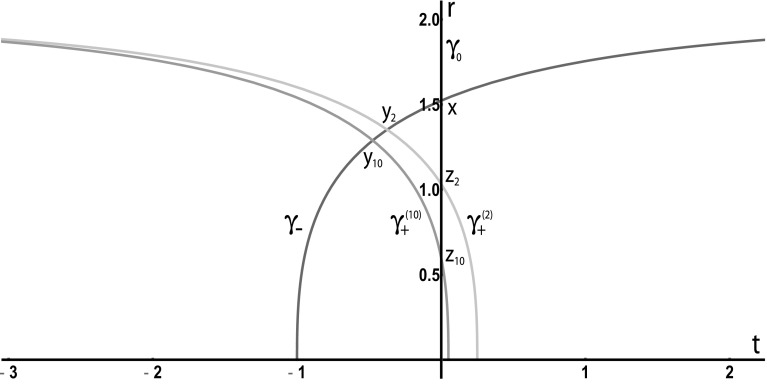
Infalling timelike geodesics for $M=1$ and $C=0.5$

Now fix the constant in $\gamma _-$ to be $-2C$ for some $C>0$ to be specified later, and denote, for $k\in \mathbb {N}$, by $\gamma ^{(k)}_+$ the pregeodesic $\gamma _+$ with constant $\frac{C}{k}$. Let *x* be the intersection of $\gamma _0$ and $\gamma _-$, $y_k$ that of $\gamma _-$ and $\gamma ^{(k)}_+$, and $z_k$ that of $\gamma ^{(k)}_+$ and $\gamma _0$ (see Fig. 6). As the time orientation in the Schwarzschild interior is directed towards $r=0$, $(x,y_k,z_k)$ is a timelike triangle with the corresponding pregeodesics from above as realizing sides.

We will use the timelike triangles $(x,y_k,z_k)$ to demonstrate that the interior Schwarzschild solution has a curvature singularity in the sense of Definition 4.20, more precisely that the timelike curvature is unbounded below.

Suppose, to the contrary, that the timelike curvature were bounded below by some $K\in \mathbb {R}$. It follows from (18) and (19) that each of the above geodesics reaches $r=0$ in finite proper time and that these finite times go to zero as $C\rightarrow 0+$. Thus, we can choose $C>0$ so small that each triangle satisfies timelike size bounds for *K*.

Consider now the scalar product of the unit tangent vectors of $\gamma _0$ and $\gamma ^{(k)}_+$ at $z_k=:(0,r_k)$. A straightforward calculation shows that this is given by

$$\begin{aligned} -\frac{1}{\sqrt{1-(t_+')^2(r_k)\left( \frac{2M}{r_k}-1\right) ^2}} \rightarrow -1 \quad (k\rightarrow \infty ), \end{aligned}$$

confirming that the triangles become degenerate, with the hyperbolic angle at $z_k$ collapsing to 0 as $k\rightarrow \infty$.

One can now apply [1, Prop. 5.1] to conclude that the comparison triangles in the model space must display the same behavior. In fact, as in [1] denote by $\gamma _{pq}$ (respectively, ${\bar{\gamma }}_{{\bar{p}} {\bar{q}}}$) the maximal timelike geodesic connecting *p* to $q\gg p$ in the spacetime (respectively, ${\bar{p}}$ to ${\bar{q}}\gg {\bar{p}}$ in $M_K$), parametrized on [0, 1]. Moreover, let $E_q(r):=\langle \gamma _{qr}'(0),\gamma _{qr}'(0)\rangle$. Then by [1, Eq. (5.3)], for a timelike geodesic triangle (*p*, *q*, *r*),

20 $$\begin{aligned} (E_q\circ \gamma _{pr})'(0) = -2 \left\langle \gamma _{pq}'(0),\gamma _{pr}'(0)\right\rangle , \end{aligned}$$

and analogously for $({\bar{p}},{\bar{q}},{\bar{r}})$ in $M_K$. The key observation now is that to calculate the left hand side of this equation, information is only required about $(E_q\circ \gamma _{pr})(t)$ for *t* arbitrarily close to 0 and that, since $p\in I^-(q)$, so is $\gamma _{pr}(t)$ for *t* small. Hence, knowledge of timelike distances suffices to determine $(E_q\circ \gamma _{pr})(t)$. More precisely, $-(E_q\circ \gamma _{pr})(t)$ equals the square of the time separation from $\gamma _{pr}(t)$ to *q*. Consequently, our assumption on timelike triangle comparison implies that $(E_q\circ \gamma _{pr})'(0)\ge ({\bar{E}}_{{\bar{q}}}\circ {\bar{\gamma }}_{{\bar{p}} {\bar{r}}})'(0)$, hence by (20) that $\langle \gamma _{pq}'(0),\gamma _{pr}'(0)\rangle \le \langle {\bar{\gamma }}_{{\bar{p}} {\bar{q}}}'(0),\gamma _{{\bar{p}} {\bar{r}}}'(0)\rangle$. Since the sidelengths of (*p*, *q*, *r*) and $({\bar{p}},{\bar{q}},{\bar{r}})$ coincide by definition, the same relation must hold between the scalar products of the unit tangent vectors.

Applying this to the timelike triangles $(x,y_k,z_k)$ and $({\bar{x}},{\bar{y}}_k,{\bar{z}}_k)$, it follows that the assumption of the lower bound *K* on the timelike curvature in the sense of Definition 4.7 implies that the hyperbolic angle at ${\bar{z}}_k$ must also go to 0 as $k\rightarrow \infty$. But in the model space $M_K$, $({\bar{x}},{\bar{y}}_k,{\bar{z}}_k)$ converges to a non-degenerate timelike triangle whose side lengths equal the (non-trivial) limits of the sidelengths of $(x,y_k,z_k)$ (hence satisfy the strict reverse triangle inequality), a contradiction.

## Classes of examples

### Continuous Lorentzian metrics

As a main application of the theory developed so far, in this subsection we are going to show that any smooth manifold endowed with a continuous, strongly causal and causally plain (as defined in [12]) Lorentzian metric provides a natural example of a Lorentzian length space.

Let *M* be a smooth manifold, and let *g* be a continuous Lorentzian metric on *M* (cf. Example 2.2). By a causal, respectively, timelike curve in *M* we mean a locally Lipschitz curve whose tangent vector is causal, respectively, timelike almost everywhere. It would also be possible to start from absolutely continuous curves, but since causal absolutely continuous curves always possess a reparametrization that is Lipschitz (cf. the discussion in [37, Sec. 2.1, Rem. 2]), the above convention is not a restriction.

The time separation function $\tau : M\times M \rightarrow [0,\infty ]$ is defined in the standard way, i.e., $\tau (x,y) = \sup \{L_g(\gamma ) : \gamma \text { future-directed causal from } x \text { to }y \}$, if $x\le y$ and $\tau (x,y)=0$ otherwise. Here, by $L_g(\gamma )$ we denote the *g*-length of a causal curve $\gamma :[a,b]\rightarrow M$, i.e., $L_g(\gamma ) = \int _a^b \sqrt{-g({\dot{\gamma }},\dot{\gamma })}\,\mathrm{d}t$. Then 2.8 (1), i.e., the reverse triangle inequality for $\tau$, just as in the smooth case, follows directly from the definition. Also, we fix any complete Riemannian metric *h* on *M* and denote by $d^h$ the metric introduced by *h*.

#### Remark 5.1

Any $L_g$-maximal curve $\gamma$ is also $L_\tau$-maximal, and $L_g(\gamma )=L_\tau (\gamma )$. In fact, suppose that $\gamma$ is future-directed causal from *p* to *q* with $L_g(\gamma )=\tau (p,q)$. Since $\tau (p,q)\ge L_\tau (\sigma )$ for any future-directed causal curve $\sigma$ from *p* to *q*, $\gamma$ is also $L_\tau$-maximal. Moreover, $L_g(\gamma )\ge L_\tau (\gamma )$ and since the converse inequality always holds (see Lemma 5.10), $L_g(\gamma )=L_\tau (\gamma )$.

#### Example 5.2

It was shown in [12] that without further assumptions, causality theory of continuous Lorentzian metrics displays a number of unexpected (and unwanted) new phenomena. Consider the following metric on $\mathbb {R}^2$ [12, Ex. 1.11]:

21 $$\begin{aligned} g = -\left( du+(1-|u|^\lambda ) dx\right) ^2 + dx^2, \end{aligned}$$

where $\lambda \in (0,1)$. Then, $g\in C^{0,\lambda }(\mathbb {R}^2)$ and *g* is smooth everywhere except on the *x*-axis. However, the light cone $J(p)\setminus I(p)$ of any point *p* on the *x*-axis has nonzero measure (and is covered by the null curves emanating from *p*). For points *q* in the interior of this region (the so-called *bubbling region*), push-up fails. Also, although $\tau (p,q)>0$, there does not exist any timelike curve connecting *p* to *q*, so $p\not \ll q$. In addition, as already noted in [22], $\tau$ is not lower semicontinuous for this spacetime: Let *p* be the origin and let $q\in \partial I^+(p)$. Then, $\tau (p,q)>0$, but taking $p_n:=(\frac{1}{n},0)$, $\tau (p_n,q)=0$ for every *n*. Consequently (fixing any background Riemannian metric *h* on $\mathbb {R}^2$), $(\mathbb {R}^2,d^h,\ll ,\le ,\tau )$ is not a Lorentzian pre-length space.

In order to exhibit additional exotic causality properties of continuous Lorentzian metrics, let us study the metric *g* from (21) in greater depth. For concreteness, set $\lambda :=\frac{1}{2}$ and let $M:=(-1,1)\times \mathbb {R}$. Thus, the metric is

$$\begin{aligned} g_{(u,x)} = -du^2 + 2 \left( \sqrt{|u|}-1\right) du\, dx + \sqrt{|u|}\left( 2-\sqrt{|u|}\right) dx^2, \end{aligned}$$

and its inverse is given by

$$\begin{aligned} g_{(u,x)}^{-1}= \sqrt{|u|}\left( \sqrt{|u|}-2\right) du^2 + 2 \left( \sqrt{|u|}-1\right) du\, dx + dx^2. \end{aligned}$$

We first collect some basic facts about the causality of (*M*, *g*), choosing the time orientation by defining $\partial _u$ to be future directed.

Let $\gamma =(\alpha ,\beta ):[a,b]\rightarrow M$ be a causal curve, then

$$\begin{aligned} 0&\ge -\dot{\alpha }(s)^2 + 2 \left( \sqrt{|u|}-1\right) \dot{\alpha }(s)\dot{\beta }(s) + \sqrt{|u|}\underbrace{\left( 2-\sqrt{|u|}\right) }_{>0}\dot{\beta }(s)^2\\&\ge -\dot{\alpha }(s)^2 + 2 \left( \sqrt{|u|}-1\right) \dot{\alpha }(s)\dot{\beta }(s), \end{aligned}$$

for all $s\in [a,b]$ such that $\dot{\gamma }(s)$ exists (i.e., for almost all $s\in [a,b]$).

Furthermore, the time orientation of $\gamma$ gives

22 $$\begin{aligned} 0&> -\dot{\alpha }(s) + \left( \sqrt{|u|} - 1\right) \dot{\beta }(s) \,(\text {if }\gamma \text { is future-directed)}, \end{aligned}$$

23 $$\begin{aligned} 0&< -\dot{\alpha }(s) + \left( \sqrt{|u|} - 1\right) \dot{\beta }(s) \,(\text {if }\gamma \text { is past-directed)}, \end{aligned}$$

again for all $s\in [a,b]$ such that $\dot{\gamma }(s)$ exists.

#### Lemma 5.3

The spacetime (*M*, *g*) is strongly causal.

#### Proof

Define $f:M\rightarrow \mathbb {R}$ by $f(u,x):=u$, then the gradient of *f* is given by $\mathrm{grad}\,(f)_{(u,x)} = \sqrt{|u|}(\sqrt{|u|}-2)\partial _u + (\sqrt{|u|}-1)\partial _x$. Thus,

$$\begin{aligned}&g_{(u,x)}\left( \mathrm{grad}\,(f), \mathrm{grad}\,(f)\right) \\&\quad = -\left( \sqrt{|u|}\left( \sqrt{|u|}-2\right) \right) ^2 + \sqrt{|u|}\underbrace{\left( \sqrt{|u|}-2\right) }_{<0}\left( \sqrt{|u|}-1\right) ^2 \le 0, \end{aligned}$$

and $g_{(u,x)}(\mathrm{grad}\,(f),\mathrm{grad}\,(f))<0$ for $u\ne 0$. Moreover, $g_{(u,x)}(\mathrm{grad}\,(f),\partial _u) = 1$, so $\mathrm{grad}\,(f)$ is past-directed causal on *M* and past-directed timelike on $(-1,0)\times \mathbb {R}$ and $(0,1)\times \mathbb {R}$, hence is a temporal function there. So strong causality holds on $(-1,0)\times \mathbb {R}$ and $(0,1)\times \mathbb {R}$.

It remains to show strong causality at points $(0,x_0)$. Let $\gamma =(\alpha ,\beta ):[a,b]$ $\rightarrow M$ be a future-directed causal curve with $\gamma (a)=(0,x_0)$ and $\gamma (b)=(u_1,x_1)$. Note that from $\mathrm{grad}\,(f)$ being past-directed causal follows that $0\le \dot{\alpha }(s)$, whenever $\dot{\gamma }(s)$ exists. So $\alpha (s)\ge 0$ for all $s\in [a,b]$ and if $u_1>0$ then $\gamma$ cannot return to a neighborhood of $(0,x_0)$ that does not contain $(u_1,x_1)$. Finally, if $\gamma$ does not enter $(0,1)\times \mathbb {R}$, then $\alpha =0$, and so by Eq. (22) we obtain $\dot{\beta }(s)>0$ almost everywhere. Consequently, $\gamma$ cannot return to a neighborhood not containing $(0,x_1)$ (note that necessarily $x_1>x_0$). $\square$

Now fix a point $q:=(u_0,x_0)$ in the (upper right) bubble region, i.e., $0< u_0 < \min (\frac{x_0}{4},1)$.

#### Proposition 5.4

There exists a maximal causal curve from 0 to *q*.

#### Proof

By Remark 5.1, it suffices to show the existence of a future-directed causal curve from 0 to *q* whose $L_g$-length is maximal. To this end, we will show that $J^+(0)\cap J^-(q)$ is compact and then refer to results from [48]. By the above we know that $J^+(0)\subseteq [0,1)\times \mathbb {R}$. The past light cone emanating from *q* is bounded by the null curves $\nu , \mu$ given below. Note that they are pregeodesics on $(0,1)\times \mathbb {R}$, since the metric is smooth there and so [4, Thm. 4.13] applies. The left bounding null curve $\nu :[0,x_0]\rightarrow M$ is given by

$$\begin{aligned} \nu (x):= {\left\{ \begin{array}{ll} \left( \frac{1}{4}\left( 2\sqrt{u_0}-x\right) ^2,\,x_0-x\right) \quad &{}\quad (x\in [0,2\sqrt{u_0}]),\\ (0,\,x_0-x)\quad &{}\quad (x\in [2\sqrt{u_0},x_0]). \end{array}\right. } \end{aligned}$$

It connects *q* to 0 and is past-directed null. Moreover, note that $\nu$ is parametrized as $x\mapsto (v(x),\,x_0-x)$ and so satisfies the equation $\dot{v}(x) = -\sqrt{|v(x)|}$ (cf. [12, Eq. (1.20)]). The other past-directed null curve emanating from *q* solves the equation

$$\begin{aligned} \dot{u}(x) = -2 + \sqrt{|u(x)|}, \end{aligned}$$

if parametrized as $\mu (x)=(u(x),\,x)$. This corresponds to the $\varepsilon =-1$ case in [12, Eq. (1.20)]. As initial condition we impose $u(x_0)=u_0$. We can solve this equation as follows: Let $F:[-1,1]\rightarrow \mathbb {R}$ be given by

$$\begin{aligned} F(s):=\int _{u_0}^s \frac{1}{-2+\sqrt{|r|}}\,\mathrm {d} r\, + x_0\quad (s\in [-1,1]). \end{aligned}$$

Then clearly $F'(s)\le -\frac{1}{2}$, and hence *F* is strictly monotonically decreasing and $F(u_0)=x_0$. Define $x':=F(0) = x_0 - \int ^{u_0}_0 \frac{1}{-2+\sqrt{|r|}}\,\mathrm {d} r$, $a:=F(1)$ and $b:=F(-1)$. Then $a<x_0<x'<b$ and $F([-1,1])=[a,b]$, so the inverse of *F* exists, $F^{-1}:[a,b]\rightarrow [-1,1]$. Finally, *u* is given as $u:=F^{-1}|_{[x_0,x']}:[x_0,x']\rightarrow (-1,1)$ and satisfies $u(x_0)=u_0$ and $u(x')=0$. Thus, $\mu :[x_0,x']\rightarrow M$, $x\mapsto (u(x),x)$ connects *q* to $(0,x')$. Then, as we saw above in Lemma 5.3, any past-directed causal curve from $(0,x')$ to 0 lies on the *x*-axis.

**Fig. 7:**
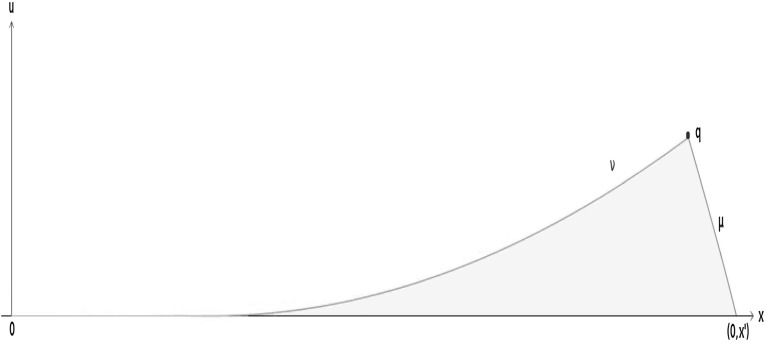
$$J^+(0)\cap J^-(q)$$ for $$q=(\frac{1}{8},1)$$

Now we see that

$$\begin{aligned}&J^+(0)\cap J^-(q) \\&\quad = \Big \{(u,x)\in M:\, x\in [0,x_0]\text { and } 0\le u \le v(x) \text{ or } x\in [x_0,x']\text { and }0\le u\le u(x)\Big \}, \end{aligned}$$

which is a compact subset of *M* (Fig. 7).

Finally, we are able conclude that ${\tilde{C}}(0,q)$ is compact (with respect to the compact-open topology) as in the first part of the proof of [48, Thm. 3.2]. So, [48, Prop. 6.4] establishes the existence of a maximal causal curve connecting 0 and *q*. $\square$

#### Corollary 5.5

Let $\gamma =(\alpha ,\beta ):[0,1]\rightarrow M$ be a maximal curve from 0 to *q*, as given by Proposition 5.4, then $\gamma$ has a branching point at which it changes its causal character (from null to timelike).

#### Proof

We first note that since the future-directed causal curve given by going from 0 along the *x*-axis to $(0,x_0)$ and then vertically to $(u_0,x_0)=q$ has length $u_0>0$, we know that $L_g(\gamma )\ge u_0 > 0$. Therefore, $\gamma$ has to be somewhere timelike and in particular, it has to leave the *x*-axis after $x_0-2\sqrt{u_0}$ (where $\nu$ intersects the *x*-axis for the first time) and before $x'$. Off the *x*-axis, i.e., on $(0,1)\times \mathbb {R}$, $\gamma$ has to be pregeodesic, since it is maximizing everywhere and the metric is smooth in this region. Consequently, $\gamma$ has a causal character on $(0,1)\times \mathbb {R}$. First, we observe that $\gamma$ cannot be null on $(0,1)\times \mathbb {R}$, since then $L_g(\gamma )$ would vanish, a contradiction. Thus, $\gamma$ has to be timelike on $(0,1)\times \mathbb {R}$. This demonstrates that $\gamma$ is a maximal causal curve whose causal character changes. Moreover, the point where it leaves the *x*-axis is a branching point as defined in Definition 4.10 because the *x*-axis itself is maximizing (and null) between any of its points. $\square$

In light of the results of Sect. 4, it can be argued that the root cause of the phenomena described above lies in the fact that the curvature of the metric *g* is unbounded near $\{u=0\}$, see [12, Eq. (1.23)].

The foregoing considerations demonstrate that Lorentzian metrics that are merely continuous do not provide a satisfactory causality theory, a problem clearly recognized already by Chrusciel and Grant [12]. As a sufficient condition for reasonable causality properties of continuous Lorentzian metrics, these authors introduced the notion of a *causally plain* Lorentzian metric. To define this concept, let us first recall from [12, Def. 1.3] that a locally Lipschitz curve $\gamma$ is called locally uniformly timelike (l.u.t.) if there exists a smooth Lorentzian metric ${\check{g}} \prec g$ such that $\check{g}({\dot{\gamma }},\dot{\gamma })<0$ almost everywhere. For $U\subseteq M$ open and $p\in U$, by ${\check{I}}^\pm (p,U)$ we denote the set of all points that can be reached by a future directed, respectively, past-directed l.u.t. curve in *U* starting in *p*. Moreover, a *cylindrical neighborhood* of a point *p* is a relatively compact chart domain containing *p* such that in this chart *g* equals the Minkowski metric at *p* and the slopes of the light cones of *g* remain close to 1 (see [12, Def. 1.8] for a precise definition). Finally, (*M*, *g*) is called causally plain if every $p\in M$ possesses a cylindrical neighborhood *U* such that $\partial {\check{I}}^\pm (p,U)=\partial J^\pm (p,U)$. Otherwise, it is called bubbling. Causally plain spacetimes in particular satisfy the standard push-up properties [12, 1.22–1.24], as well as $I^\pm (p)={\check{I}}^\pm (p)$ for every $p\in M$. Any spacetime with a Lipschitz continuous metric is causally plain [12, Cor. 1.17].

Our main aim in this section is to establish that $(M,d^h,\ll ,\le ,\tau )$ as defined above is a Lorentzian length space for any continuous, strongly causal and causally plain metric *g*.

#### Lemma 5.6

Let (*M*, *g*) be causally plain, and let $p,q\in M$. Then, $p\ll q$ if and only if $\tau (p,q)>0$.

#### Proof

That $p\ll q$ implies $\tau (p,q)>0$ is immediate from the definition of $\tau$.

For the converse implication, suppose that $\tau (p,q)>0$ and let $\gamma$ be a future-directed causal curve from *p* to *q* with $L_g(\gamma )\ge \frac{1}{2}\tau (p,q)>0$. If we can show that $\gamma$ enters $I^+(p)$ then $p\ll q$ will follow from push-up [12, Lemma 1.22]. Since the length of $\gamma$ is strictly positive, the restriction of $\gamma$ to a suitable subinterval will also have positive length while at the same time being contained in a cylindrical neighborhood as in the definition of causally plain spacetimes around some point on $\gamma$. So without loss of generality we may suppose that $\gamma$ is contained in such a neighborhood *U* around *p*. Now let us suppose that $\gamma$ never enters $I^+(p,U)$, i.e., that it remains within $J^+(p,U)\setminus I^+(p,U)$. By our assumption on *U*, [12, Prop. 1.10 (v)] implies that $\partial J^+(p,U) = \partial {\check{I}}(p,U) = \partial I(p,U)$ is given (in the cylindrical chart over *U*) as the graph of a Lipschitz function *f*. It follows that $\gamma$ lies entirely within the graph of *f*. But then the proof of [12, Prop. 1.21 (v)] shows that ${\dot{\gamma }} (t)$ (if it exists) cannot be *g*-timelike at any *t*. This contradicts the fact that $L_g(\gamma )>0$. $\square$

#### Proposition 5.7

Let (*M*, *g*) be causally plain. Then, the time separation function $\tau : M\times M \rightarrow [0,\infty ]$ is lower semicontinuous.

#### Proof

We basically follow [4, Lemma 4.4]. Let $p,q\in M$. If $\tau (p,q)=0$, there is nothing to prove. Next, let $0<\tau (p,q)<\infty$ and, given any $0<\epsilon < \tau (p,q)$, choose a future-directed causal curve $\gamma : [0,1]\rightarrow M$ from *p* to *q* with $L_g(\gamma )\ge \tau (p,q)-\frac{\varepsilon }{2}$. Now pick $0<t_1<t_2<1$ such that $0<L_g(\gamma |_{[0,t_1]})<\frac{\varepsilon }{4}$ and $0<L_g(\gamma |_{[t_2,1]})<\frac{\varepsilon }{4}$. Set $p_1:=\gamma (t_1)$ and $q_1:=\gamma (t_2)$, as well as $U:=I^-(p_1)$ and $V:=I^+(q_1)$. Since $L_g(\gamma |_{[0,t_1]})>0$, $\tau (p,p_1)>0$, which by Lemma 5.6 implies that $p\in I^-(p_1)$. Analogously, $V:=I^+(q_1)$ is an open neighborhood of *q*. For any $(p',q')\in U\times V$ we obtain

$$\begin{aligned} \begin{aligned} \tau (p',q')&\ge \tau (p',p_1) + \tau (p_1,q_1) + \tau (q_1,q') \ge L_g(\gamma |_{[t_1,t_2]})\\&= L_g(\gamma ) - L_g(\gamma |_{[0,t_1]}) - L_g(\gamma |_{[t_2,1]}) \ge \tau (p,q) - \varepsilon , \end{aligned} \end{aligned}$$

and thereby lower semicontinuity of $\tau$ at (*p*, *q*).

Finally, if $\tau (p,q)=\infty$, there are causal curves of arbitrary length from *p* to *q*. Then, the previous argument shows that the same is true of any points $p'$, $q'$ in *U*, respectively, *V*. $\square$

Collecting the previous results, we obtain:

#### Proposition 5.8

Let (*M*, *g*) be a spacetime with a continuous causally plain metric. Then, $(M,d^h,\ll ,\le ,\tau )$ is a Lorentzian pre-length space.

In order for a spacetime with a continuous causally plain metric to give rise to a Lorentzian length space, there is one further requirement, namely we have to make sure that the notions of causal curve and of *R*-causal curve coincide. In fact, as our next result will show, this is guaranteed if the spacetime is strongly causal. In its proof, we will make use of *time functions*, i.e., functions that increase along any future-directed causal curve. In any continuous spacetime, there always exist smooth local time functions (e.g., the time coordinate in any cylindrical neighborhood).

#### Proposition 5.9

For a continuous and strongly causal spacetime (*M*, *g*), the notions of causal and R-causal curve coincide.

#### Proof

By [40, Lemma 3.21] (which remains valid for continuous spacetimes), strong causality implies that any point in *M* possesses a neighborhood basis consisting of causally convex neighborhoods (i.e., such that any causal curve with endpoints contained in the neighborhood remains entirely within it). Suppose now that $\gamma :I \rightarrow M$ is an R-causal curve and let $t_0\in I$. By [10, Lemma 15] it suffices to show that for any smooth local time function *f* in a neighborhood *U* of $\gamma (t_0)$, $f\circ \gamma$ is non-decreasing near $t_0$. To this end, let $V\subseteq U$ be causally convex and let $J\subseteq I$ be an open interval around $t_0$ such that $\gamma (J)\subseteq V$. Then by R-causality, for $t_1<t_2$ in *J* there exists a future-directed causal curve $\sigma$ in *M* (and consequently in *V*) from $\gamma (t_1)$ to $\gamma (t_2)$. By definition, *f* is non-decreasing along $\sigma$, so in particular $f(\gamma (t_1))\le f(\gamma (t_2))$. $\square$

Adding the assumption of strong causality, and defining ${\mathcal {T}}$ as in Definition 3.22, we have:

#### Lemma 5.10

Let (*M*, *g*) be a causally plain spacetime with a continuous, strongly causal metric and let $p,q\in M$. Then, $\tau (p,q)={\mathcal {T}}(p,q)$. Moreover, for any causal curve $\gamma$, $L_g(\gamma )\le L_\tau (\gamma )$.

#### Proof

Note first that the claimed inequality on the lengths of causal curves follows from the first part of the proof of Proposition 2.32.

Also, ${\mathcal {T}}(p,q)\le \tau (p,q)$ was already shown in Remark 3.23 (iii).

To show the converse inequality suppose, to the contrary, that there exists some $\epsilon >0$ such that ${\mathcal {T}}(p,q)+\varepsilon < \tau (p,q)$. Then, by the definitions of ${\mathcal {T}}$ and $\tau$ there exists some future-directed causal curve $\sigma$ from *p* to *q* such that for any future-directed causal curve $\gamma$ we have

$$\begin{aligned} L_\tau (\gamma )< \tau (p,q) - \varepsilon < L_g(\sigma ) \le L_\tau (\sigma ). \end{aligned}$$

Setting $\gamma :=\sigma$ now gives a contradiction. $\square$

#### Remark 5.11

(i) This result indicates that the length coming from a time separation function $\tau$ that in turn is defined via a length functional, is better behaved than $L_\tau$ for a generic $\tau$. In fact, in Lemma 5.10 we show $\tau = {\mathcal {T}}$ without knowing whether $L_\tau$ equals $L_g$.
(ii) The assumption of causal plainness in Lemma 5.10 is in fact not needed for the proof. However, it was added as otherwise $\tau$ would not be lower semicontinuous in general and hence one would not obtain a Lorentzian pre-length space.

Based on this, we can proceed to proving the main result of this section:

#### Theorem 5.12

Let (*M*, *g*) be a spacetime with a continuous, strongly causal and causally plain metric. Then, $(M,d^h,\ll ,\le ,\tau )$ is a strongly localizable Lorentzian length space.

#### Proof

Due to Proposition 5.8, Proposition 3.5, Lemma 5.10 and the fact that spacetimes are causally path connected by definition, it remains to establish that $(M,d^h,\ll ,\le ,\tau )$ is strongly localizable (Definition 3.16). We first note that by Proposition 5.9 *g*-causal curves are the same as causal curves in the sense of Definition 2.18; hence, we may speak of causal curves without ambiguity.

Let $p\in M$, then there is a neighborhood *U* of *p* such that the *h*-arclength of all causal curves in *U* is bounded by some constant $C>0$ by [13, Lemma 2.6.5] or [22, Lemma 2.1]. This gives 3.16 (i).

At this point, let ${\hat{g}}$ be a smooth Lorentzian metric on *M*, with $g\prec {\hat{g}}$ (see [12]). Then, there exists an (arbitrarily small) ${\hat{g}}$-globally hyperbolic neighborhood $(V, x^\mu )$ of *p* that is causally convex in *U* by [40, Thm. 2.14] (cf. also [51, Thm. 2.2]). This means that in the $x^\mu$-coordinates one has that $x^0=0$ is a Cauchy hypersurface in *V* with respect to ${\hat{g}}$ and that any ${\hat{g}}$-causal curve in *U* with endpoints in *V* is contained in *V*. Then, $x^0=0$ is also a Cauchy hypersurface with respect to *g*; hence, $(V,g|_V)$ is globally hyperbolic by [48, Thm. 5.7], and thus maximal (in *V*) causal curves exist between any two (in *V*) causally related points by the Avez–Seifert result for continuous metrics [48, Prop. 6.4]. By strong causality, we can without loss of generality assume that *V* is actually causally convex in *M*. Clearly, $I^\pm (x)\cap V \ne \emptyset$ for every $x\in V$.

Now define $\omega :V\times V\rightarrow [0,\infty )$ by $\omega (x,y):=\tau (x,y)$ for $x,y\in V$. Note that any causal curve from $x\in V$ to $y\in V$ is contained in *V*, hence $\tau (x,y)<\infty$ as there exists a maximal causal curve. This curve is actually globally maximal since *V* is causally convex in *M*. This also implies that $\omega$ is continuous. It is lower semicontinuous by Proposition 5.7, so assume that it were not upper semicontinuous at $(x,y)\in V\times V$. Thus, there is a $\delta >0$ and sequences $x_n\rightarrow x$, $y_n\rightarrow y$ in *V* such that

$$\begin{aligned} \tau (x_n,y_n)\ge \tau (x,y)+\delta , \end{aligned}$$

which implies $\tau (x_n,y_n)>0$. Consequently, there is a future-directed causal curve $\alpha _n$ from $x_n$ to $y_n$ with $L_g(\alpha _n) > \tau (x_n,y_n)-\frac{1}{n}$. Since *V* is globally hyperbolic there is a limit curve $\alpha$ from *x* to *y* of the $\alpha _n$s (by the limit curve theorem [35]), that satisfies $L_g(\alpha )\ge \tau (x,y)+\delta$—a contradiction. This shows that $\omega$ is continuous and establishes 3.16 (ii).

By the above we have that for any $x,y\in V$ with $x<y$ there is a globally maximal causal curve $\gamma _{x,y}$ from *x* to *y*. Thus, $\gamma _{x,y}$ is also $\tau$-maximal by Remark 5.1 and so $L_\tau (\gamma _{x,y}) = \tau (x,y) = \omega (x,y)$, which establishes 3.16 (iii). $\square$

#### Example 5.13

There are large classes of spacetimes that are in fact regular Lorentzian length spaces, namely:

(i) Strongly causal Lorentzian metrics *g* of regularity at least $C^{1,1}$. Indeed, for such metrics property (iv) from Definition 3.16 is an immediate consequence of the Gauss Lemma (cf. [36] or [30]).
(ii) Continuous causally plain and strongly causal Lorentzian metrics whose causal curvature is bounded above. In fact, in this case Definition 3.16 (iv) is satisfied by Remark 4.16.

### Closed cone structures

Many results from smooth causality theory can be generalized to cone structures on smooth manifolds. The interest in such generalizations originated in the problem of constructing smooth time functions in stably causal or globally hyperbolic spacetimes. Fathi and Siconolfi [18] studied continuous cone structures and employed methods from weak KAM theory to address this problem. In [10], Bernard and Suhr considered closed cone structures and developed a theory of Lyapunov functions for such cone structures, showing, among other results, the equivalence between stable causality and the existence of temporal functions, or between global hyperbolicity and the existence of steep temporal functions in this setting. The deepest and most comprehensive study of causality theory for closed cone structures to date is the very recent work by Minguzzi [37]. It provides a complete causality theory, establishing the full causal ladder for such cone structures and contains manifold applications, among others to time and temporal functions, singularity theorems, embedding of Lorentzian manifolds into Minkowski spacetime and non-commutative geometry.

In this section, we follow the approach in [37] and show that closed cone structures provide a rich source of examples of Lorentzian pre-length and length spaces. We begin by recalling some basic definitions.

A *sharp cone* in a vector space *V* is a subset of $V\setminus \{0\}$ that is positively homogeneous, closed in the trace topology of *V* on $V\setminus \{0\}$, convex, and does not contain any line through 0. It is called proper if its interior is non-empty. A *cone structure* on a smooth manifold *M* is a map $x\mapsto C_x$ that assigns to each $x\in M$ a sharp non-empty cone. The cone structure is called *closed* if it forms a closed subbundle of the slit tangent bundle of *M*. It is called *proper* if it is closed and $\mathrm {int}(C)_x\not =\emptyset$ for each $x\in M$. (Semi-)continuity of cone structures is formulated in terms of the Hausdorff distance on local sphere bundles, see [18, Sec. 2], [37, Sec. 2].

An absolutely continuous curve $\gamma$ in *M* is called causal for the cone structure *C* if ${\dot{\gamma }}(t)\in C_{\gamma (t)}$ almost everywhere. Timelike curves are by definition piecewise $C^1$-solutions of the differential inclusion ${\dot{\gamma }}(t)\in \mathrm {int}(C)_{\gamma (t)}$. Based on these notions, one defines the chronological and causal relations $\ll$ and < as usual, whereby any locally Lipschitz cone structure induces a causal space in the sense of Definition 2.1 (cf. [37, Thm. 8]).

The following notions were introduced in [37, Sec. 2.13]: Given a closed cone structure (*M*, *C*) and a concave, positively homogeneous function ${\mathcal {F}}: C\rightarrow [0,\infty )$, a cone structure on $M^\times := M\times \mathbb {R}$ is defined by

$$\begin{aligned} C^\times _{(p,r)} := \left\{ (y,z) : y\in C_p, |z|\le {\mathcal {F}}(y)\right\} . \end{aligned}$$

The corresponding cone structure $(M^\times ,C^\times )$ is called a *Lorentz–Finsler space* $(M,{\mathcal {F}})$. The latter is called closed, respectively, proper, respectively locally Lipschitz if $(M^\times ,C^\times )$ has these properties. On a closed Lorentz–Finsler space, the length of a causal curve $\gamma :[0,1]\rightarrow M$ is defined by $L(\gamma ):=\int _0^1 {\mathcal {F}}({\dot{\gamma }})\,\mathrm{d}t$. The corresponding *Lorentz–Finsler distance* is defined as $\tau (p,q)= 0$ if $p\not \le q$, and $\tau (p,q):=\sup _\gamma L(\gamma )$ otherwise, where the supremum is taken over all future-directed causal curves from *p* to *q*. Finally, fix a complete Riemannian metric *h* on *M*. Then, we have:

#### Proposition 5.14

Let $(M,{\mathcal {F}})$ be a locally Lipschitz proper Lorentz–Finsler space such that ${\mathcal {F}}(\partial C)=0$. Then, $(M,d^h,\ll ,\le ,\tau )$ is a Lorentzian pre-length space.

#### Proof

In fact, under these assumptions $\tau$ is lower semicontinuous by [37, Thm. 52]. Furthermore, $\tau (p,q)>0\Leftrightarrow p\ll q$ follows from [37, Thm. 55]. The other properties are immediate from the definitions. $\square$

As in the case of continuous spacetimes, if we want to proceed to establishing the properties of Lorentzian length spaces we first have to secure that the classes of R-causal and causal curves coincide. In fact, this is true for any strongly causal closed cone structure, hence in particular for any strongly causal proper Lorentz–Finsler space: Since the existence of arbitrarily small causally convex neighborhoods in this case holds by definition, this follows exactly as in the proof of Proposition 5.9. Moreover, defining ${\mathcal {T}}$ as in Definition 3.22, the same proof as in Lemma 5.10 gives:

#### Lemma 5.15

Let $(M,{\mathcal {F}})$ be a locally Lipschitz proper Lorentz–Finsler space such that ${\mathcal {F}}(\partial C)=0$ and suppose that $(M,{\mathcal {F}})$ is strongly causal. Then, for all $p, q \in M$, ${\mathcal {T}}(p,q)=\tau (p,q)$.

Finally, we can prove:

#### Theorem 5.16

Let $(M,{\mathcal {F}})$ be a locally Lipschitz proper Lorentz–Finsler space such that ${\mathcal {F}}(\partial C)=0$. If $(M,{\mathcal {F}})$ is strongly causal, then $(M,d^h,\ll ,\le ,\tau )$ is a strongly localizable Lorentzian length space.

#### Proof

By strong causality and [37, Prop. 7], any point *x* in *M* possesses a basis of open neighborhoods that are globally hyperbolic and causally convex. We may therefore pick such a neighborhood $\Omega _x$ such that $\Omega _x$ is causally closed. Since *M* is causally path connected by definition and taking into account Lemma 5.15, it only remains to establish strong localizability, i.e., properties (i)–(iii) from Definition 3.16 for a neighborhood basis. Now for any $\Omega _x$, (i) follows from [37, Prop. 7], and to obtain (ii), by [37, Thm. 52 and Thm. 58], we may set $\omega _x:=\tau |_{\Omega _x\times \Omega _x}$. Finally, (iii) follows from the Avez–Seifert theorem [37, Thm. 54] (or also from Theorem 3.30). $\square$

Moreover, just as in Example 5.13 (ii), regularity and (SR)-localizability can be achieved by assuming upper causal curvature bounds.

Finally, a natural open question is whether one can weaken the assumption of local Lipschitz continuity of the cone structure by an analogue of causal plainness, as in the case of continuous spacetimes.

### Outlook on further examples

The framework developed in the previous sections makes it possible to handle situations where one might not have the structure of a manifold or Lorentz(-Finsler) metric. Even in these cases, the theory of Lorentzian (pre-) length spaces allows one to define timelike and causal curvature (bounds) via triangle comparison. Thus, it provides a new perspective on curvature in such cases where there is no classical notion of curvature (Riemann tensor, Ricci and sectional curvature, etc.). This is applies, in particular, to certain approaches to quantum gravity, as pointed out in, e.g., [38] (see also the corresponding paragraph in the introduction, Sect. 1). In this non-rigorous outlook, we briefly sketch two such approaches, namely *causal Fermion systems* [16, 17] and the theory of causal sets [6].

The underlying idea in both cases is that the structure of spacetime has to be modified on a microscopic scale to include quantum effects. This gives rise to non-smoothness of the underlying geometry, and only in the macroscopic picture the classical spacetime (i.e., a Lorentzian manifold) emerges. We briefly recall the relevant definitions, the causal structures and discuss the connections to Lorentzian (pre-)length spaces.

We start with the recent approach of causal Fermion systems. Let *H* be a separable complex Hilbert space, and let $n\in \mathbb {N}$. Let $F\subseteq L(H)$ be the set of all self-adjoint operators on *H* of finite rank that have at most *n* positive and *n* negative eigenvalues. Let $\rho$ be a measure defined on a $\sigma$-algebra of subsets of *F*, called the *universal measure*. Then, $(H,F,\rho )$ is called a *causal Fermion system*. The *spacetime* *M* is defined as $M:=\mathop {\mathrm {supp}}(\rho )\subseteq F$, the support of the universal measure $\rho$, with the induced topology from *L*(*H*). The causal structure arises as follows: For $x,y\in M$ the product $x y:=x\circ y$ has at most 2*n* eigenvalues. If all of them have the same absolute value, *x* and *y* are called *spacelike* separated. If they do not all have the same absolute value and are real, then *x* and *y* are called *timelike* separated. In all other cases, *x* and *y* are called *lightlike* separated. There is also a notion of time orientation as follows: For an operator $x\in M$ denote by $\pi _x$ the orthogonal projection on the subspace *x*(*H*). Define the anti-symmetric function $C:M\times M\rightarrow \mathbb {R}$ by $C(x,y):= i\, \mathrm {tr}(y x \pi _y \pi _x - x y \pi _x \pi _y)$. One can therefore define that *y lies to the future (past) of x* if $C(x,y)>0$ ($C(x,y)<0$, respectively). Now one can define *timelike and causal chains* and the causal relations $x\ll y$ if there is (a future-directed) timelike chain from *x* to *y* and analogously $x<y$ for causal chains. At this point, one is able to introduce a *Lorentzian distance* on *M* and obtain the structure of a Lorentzian pre-length space, as described in [17, Subsec. 5.1]. Whether this gives the structure of a Lorentzian length space will be considered elsewhere.

Another approach to quantum gravity is the theory of *causal sets*, which is closely related to Lorentzian pre-length spaces and Example 2.16.

Let $(X,\le )$ be a partially ordered set that is locally finite, i.e., for every $x,y\in X$ the set $J(x,y):=\{z\in X: x\le z\le y\}$ is finite. Writing $x<y$ if $x\le y$ and $x\ne y$ we define $I(x,y):=\{z\in X: x<z< y\}$. This minimal framework induces an analogous notion of geodesics or maximal curves as follows: $(x,y)\in X^2$ is called a *link* if $x\le y$ and $I(x,y)=\emptyset$. A *chain* is a sequence of points $(x_i)_{i=1}^n$ with $x_i<x_{i+1}$ for $i=1,\ldots ,n-1$, and moreover, a chain is a *path* if every pair $(x_i,x_{i+1})$ is a link. The length of a chain $C=(x_i)_{i=1}^n$ is $l(C)=n$ and a *geodesic* between *x* and *y* (for $x<y$) is a path from *x* to *y* whose length is maximal over all paths from *x* to *y*.

To include causal sets in the framework of Lorentzian pre-length spaces, define $\ll :=<$, i.e., $x\ll y$ if and only if $x<y$. Then, $(X,\ll ,\le )$ is a causal space (in the sense of Definition 2.1) and we define $\tau (x,y):=\sup \{l(C): C$ is a chain from *x* to $y\}$ and $\tau (x,y)=0$ if there is no chain from *x* to *y*. Putting any metric *d* on *X* that makes $\tau$ lower semicontinuous yields a Lorentzian pre-length space $(X,d,\ll ,\le ,\tau )$. Since *J*(*x*, *y*) is finite for every $x,y\in X$ there is no metric on *X* that allows continuous parametrizations of *J*(*x*, *y*) and thus it cannot be turned into a Lorentzian length space. However, there is a close connection and it seems possible to discretize a Lorentzian length space as one can discretize a Lorentzian manifold to obtain a causal set.
